# Reduced expression of innate immunity-related genes in lymph node metastases of luminal breast cancer patients

**DOI:** 10.1038/s41598-021-84568-0

**Published:** 2021-03-03

**Authors:** Marta Popeda, Aleksandra Markiewicz, Tomasz Stokowy, Jolanta Szade, Magdalena Niemira, Adam Kretowski, Natalia Bednarz-Knoll, Anna J. Zaczek

**Affiliations:** 1 Laboratory of Translational Oncology, Intercollegiate Faculty of Biotechnology, Medical University of Gdansk, 80-211 Gdansk, Poland; 2 Department of Clinical Science, University of Bergen, 5021 Bergen, Norway; 3 Department of Pathomorphology, Medical University of Gdansk, 80-211 Gdansk, Poland; 4 Clinical Research Centre, Medical University of Bialystok, 15-276 Bialystok, Poland

**Keywords:** Breast cancer, Metastasis

## Abstract

Immune system plays a dual role in cancer by either targeting or supporting neoplastic cells at various stages of disease, including metastasis. Yet, the exact immune-related transcriptome profiles of primary tumours (PT) and lymph node metastases (LNM) and their evolution during luminal breast cancer (BCa) dissemination remain undiscovered. In order to identify the immune-related transcriptome changes that accompany lymphatic spread, we analysed PT-LNM pairs of luminal BCa using NanoString technology. Decrease in complement C3—one of the top-downregulated genes, in LNM was validated at the protein level using immunohistochemistry. Thirty-three of 360 analysed genes were downregulated (9%), whereas only 3 (0.8%) upregulated in LNM when compared to the corresponding PT. In LNM, reduced expression was observed in genes related to innate immunity, particularly to the complement system (*C1QB*, *C1S*, *C1R*, *C4B*, *CFB*, *C3*, *SERPING1* and *C3AR1*). In validation cohort, complement C3 protein was less frequently expressed in LNM than in PT and it was associated with worse prognosis. To conclude, local expression of the complement system components declines during lymphatic spread of non-metastatic luminal BCa, whilst further reduction of tumoral complement C3 in LNM is indicative for poor survival. This points to context-dependent role of complement C3 in BCa dissemination.

## Introduction

The metastatic disease remains the leading cause of cancer-related deaths. Metastasis is a multistep process that involves the action of both tumour microenvironment (TME), comprising immune cells and stromal components, and cancer cells. The current opinion states that cancer cells tend to spread either via lymph or blood, reaching their specific final destination—lymph nodes or distant organs^1^. In breast cancer (BCa), lymph nodes are the first site to be colonized through the lymphatic route, usually much earlier than the distant sites reached via haematogenic route. Recent research has demonstrated that distant metastases of BCa may be seeded from the metastatic foci in lymph nodes^2–4^. As lymph nodes play a key role in immune response, they may also contribute to the selection of the immune-evading phenotype of cancer cells, thus driving further metastatic spread. Still, the transcriptional changes that accompany the dissemination process remain unknown.

The immune system, although originally developed for defence against pathogens, is a key player in cancer development and progression. The interaction between the tumour and surrounding immune cells is constant and complex, leading either to inhibition or stimulation of tumour growth, as included in the hallmarks of cancer by Hanahan and Weinberg^5^. In contrary to common knowledge, luminal breast tumours have recently been demonstrated to exhibit heterogeneous immunogenicity reflected by distinct patterns of immune gene expression^6,7^.

Thus, in this study we aimed to explore the changes of immune-related transcriptome indicative for metastatic colonization in luminal BCa. We compared data on 360 immune-related genes expression in matched pairs of primary breast tumours (PT) and lymph node metastases (LNM). To compensate for the physiological differences between breast and lymph node tissue, we incorporated a healthy background normalization step based on normal tissue expression data from GeneCards database. The selected transcriptional changes were subsequently validated at the protein level using immunohistochemical (IHC) staining.

## Results

### Immune-related transcriptomic changes during metastatic colonization in luminal BCa

#### LNM vs. LYMPH NODE comparison

**Figure 1:**
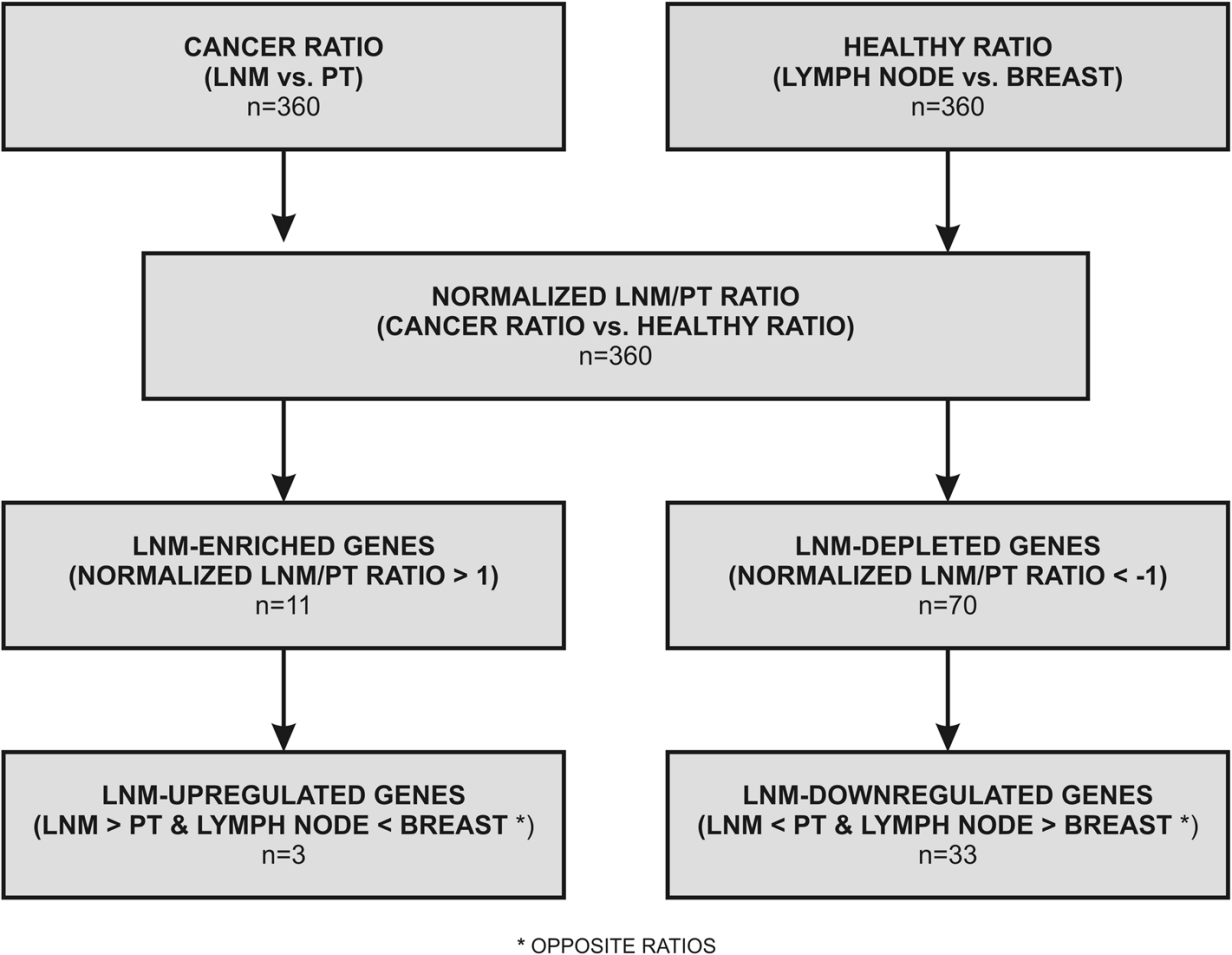
General flow of data analysis. 360 genes were analysed in 11 matched pairs of PT and LNM tissues (CANCER; NanoString data) with a healthy-background normalization based on expression data for healthy breast and lymph node tissues (HEALTHY; Illumina Body Map data); for each gene, normalized LNM/PT ratio was calculated for each matched LNM-PT pair separately, and the median of all normalized LNM/PT ratios was employed in further analyses.

Owing to the physiological transcriptome differences between lymph node and breast tissue, we decided to include a healthy-background-normalization step in our analysis (Fig. 1). Using expression data for lymph nodes (LYMPH NODE) and breast tissue (BREAST) from GeneCards database (GTEx project data^8^), we calculated median normalized LNM/PT ratios for 360 genes linked with the immune system.

Here we observed a substantial disproportion in the number of genes that were enriched or depleted in LNM in comparison to healthy lymph node. Eleven of 360 genes demonstrated increased expression in LNM, while a decrease was observed in 70 genes (Supplementary Table S1). LNM-enriched genes were primarily breast, breast cancer or pro-metastatic markers, while the LNM-depleted genes mainly contributed to innate immune response. These observations showed that in the LNM, the healthy lymphatic tissue—physiologically abundant in immune-related transcripts—was replaced by cancer, which putatively induced changes in stromal cells (TME) and was connected with decreased innate immune response.

#### LNM versus PT comparison

**Figure 2:**
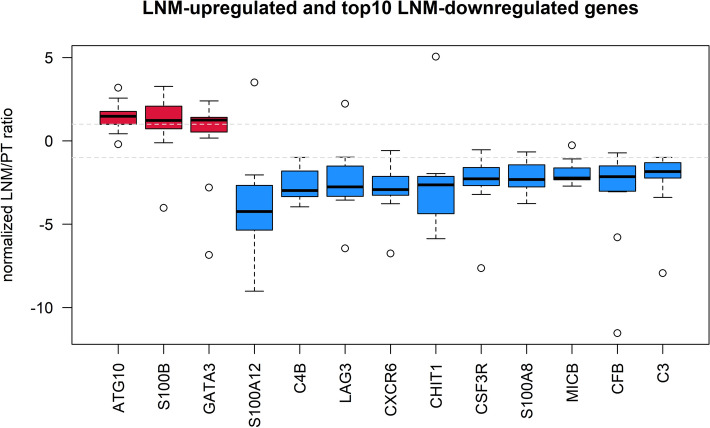
Distribution of normalized LNM/PT ratios of LNM-upregulated and -downregulated genes. Genes were classified as LNM-upregulated (red) and LNM-downregulated (blue; top10 out of 33 genes are depicted) based on the median normalized LNM/PT ratio and opposite LYMPHNODE/BREAST and median matched LNM/PT ratios. Grey dashed lines represent the cut-off for LNM-enrichment (1) and LNM-depletion (−1).

In the next step, we looked for transcriptional changes from PT to LNM. Based on the opposite ratios of gene expression levels in LYMPH NODE/BREAST and LNM/PT, we selected LNM-upregulated genes from LNM-enriched genes (3/11) and LNM-downregulated genes from LNM-depleted genes (33/70) (Supplementary Table S1). All LNM-upregulated and top10 LNM-downregulated genes are depicted in Fig. 2.

**Figure 3:**
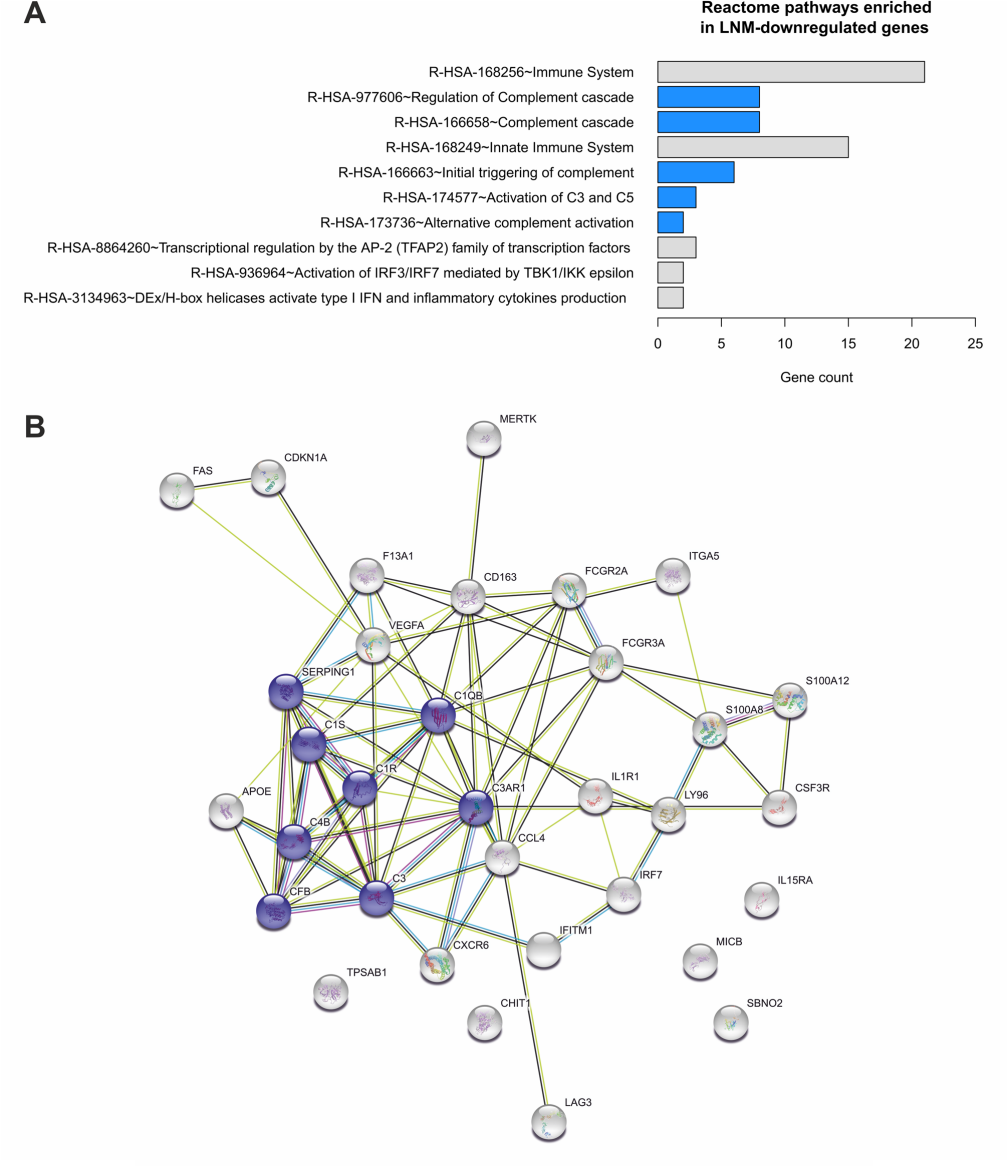
LNM-downregulated genes are associated with the complement system. (**A**) ConsensusPathDB over-representation analysis [pathways ordered according to − log 10(FDR adjusted *p* value), complement-related pathways marked in blue] and (**B**) STRING protein interaction analysis (complement-related protein products marked in purple).

Considering their biological role, 3 LNM-upregulated genes (*ATG10*, *GATA3* and *S100B)* are potential markers of aggressive phenotype and increased metastatic potential of cancer cells. On the other hand, 33 LNM-downregulated genes are mainly associated with innate immunity, in particular, with the complement cascade, as revealed by the functional annotation analysis (Fig. 3, Supplementary Table S2).

### Complement component 3 (C3) protein expression decreases during metastasis—IHC validation

Downregulation of transcripts related to complement cascade in LNM, when compared to corresponding PT, indicates that local expression of complement might play a distinct role in PT and LNM. For that reason we decided to validate our transcriptional findings at the protein level, focusing on the central element of all activation cascades—complement component 3 (C3). Using IHC staining, we evaluated C3 expression in the larger cohort of luminal BCa patient (n = 79) with both positive (N+, n = 43) and negative (N−, n = 36) nodal involvement status, including the samples analysed with NanoString technology.

**Figure 4:**
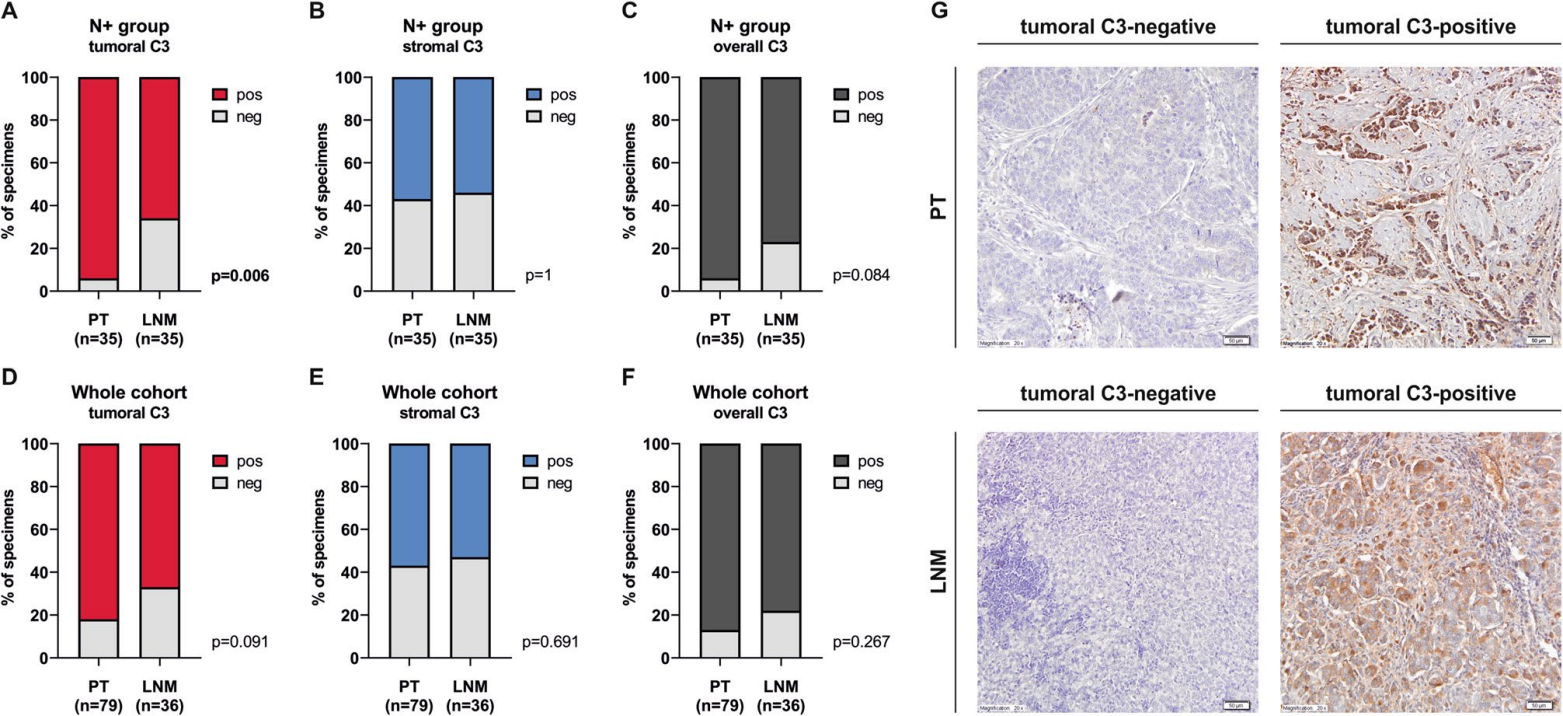
C3 protein expression is reduced during the lymphatic spread of BCa. N+ group evaluated for tumoral (**A**), stromal (**B**) and overall (**C**) C3 expression in PT and LNM; whole cohort evaluated for tumoral (**D**), stromal (**E**) and overall (**F**) C3 expression in PT and LNM; number of informative specimens is presented in Supplementary Figure S4; distribution was estimated with Fisher’s exact test. Representative images of C3 immunohistochemical staining in PT and LNM (**G**); presented tumoral C3-positive tissues also show a weak stromal C3 expression.

The C3 staining was informative for 36 of 36 N− PT, 42 of 43 N+ PT and 36 of 43 LNM specimens, resulting in 35 matched PT-LNM pairs. We evaluated stromal, tumoral and overall (i.e. combined C3 expression in both tumour cells and stroma) C3 expression status and dichotomized it as negative or positive based on staining intensity. In 11 matched PT-LNM pairs for which both NanoString and IHC data were available, mRNA levels and protein status tended to correlate when C3 protein was evaluated in tumour cells, however, did not reach the statistical significance due to the low number of samples (Supplementary Figure S2). In addition, in N+ patients, tumoral C3 was significantly decreased in LNM in comparison to PT (Fig. 4A), while no difference in C3 presence was noted in the stromal compartment (Fig. 4B). Combined stromal and tumoral assessment (overall status) showed a trend toward reduced C3 positivity in LNM (Fig. 4C). Similar tendencies were also observed when N+ and N− patients were analysed together (Fig. 4D–F). Representative images of tumoral C3 staining are presented in Fig. 4G. Of note, C3 expression in tumour cells of PT did not correlate with any clinical features, including stage, T status, grade and CTC status (data not shown). Intriguingly, loss of C3 expression in LNM tumour cells was associated with shorter 3-year overall survival (Supplementary Figure S3).

## Discussion

Interactions between tumour cells and TME remain still unexplored, particularly in the context of cancer progression. In the current study, we focused on the most common molecular subtype of BCa—luminal cancers, where we demonstrate that the immune-related transcriptome changes occurring between primary tumour (PT) and lymph node metastasis (LNM) are mainly related to innate immunity, i.e. complement pathway.

In the exploratory group consisting of matched PT and LNM from 11 non-metastatic luminal BCa patients, 9% of transcripts were downregulated, whereas only 0.8% of transcripts were upregulated in LNM when compared to PT. According to the literature, all 3 identified LNM-upregulated genes, i.e. *ATG10*, *GATA3* and *S100B*, are considered markers of breast cancer cells and their invasive potential^9–13^. *ATG10* encodes a protein involved in autophagy cascade known to support or even induce metastatic process^9,10^. *GATA3* codes for a transcription factor that is particularly abundant in luminal epithelial cells and directly correlates with *ESR1* expression, thus is considered a marker of luminal breast cancer^11,12^, whereas *S100B* belongs to a family of genes coding for calcium-binding inflammatory proteins, regulators of p53, and might be involved in acid-induced EMT of ER-positive breast cancer cells in vitro^14^. The above-described enrichment in breast cancer and metastasis-associated transcripts proves that our algorithm is capable of adjusting the detected changes to the original characteristics of compared tissues, presumably due to the appropriate application of healthy tissue normalization step. On the other hand, the genes that were downregulated in LNM in comparison to PT were mainly associated with innate immunity, in particular complement system pathways. Interestingly, the identified complement-related genes are implicated in the early steps of the cascade, including all 3 pathways of its activation—*C1QB*, *C1S*, *C1R*, *C4B*, *CFB* and *C3*, regulation—*SERPING1*, as well as its effector mechanisms—*C3AR1* (coding for receptor expressed on the surface of a variety of immune cells).

The complement system is known to play a dual role in cancer. As a fundamental part of the innate immunity, it is capable of targeting cancer cells and managing the immune response against the tumour. On the other hand, as a potent pro-inflammatory mechanism, the complement system is thought to substantially contribute to tumour growth by generating chronic inflammation state that facilitates mobilization of immune suppressor cells^15^ and supports angiogenesis^16^. In general, proteins comprising the complement system are synthesized in the liver and then released into plasma, resulting in the extracellular body compartments being the main environment for their interaction and cascade activation^17–20^. Nonetheless, a growing body of evidence suggests that the individual components of the complement pathway may also be produced within a tumour by both cancer and stromal cells. This is also supported by our data on high expression of C3 protein in tumour cells in BCa. Still, the locally produced complement proteins are thought to have a non-canonical function and act in a context-dependent manner, which merits further exploration^18,21,22^.

The association between the complement system and tumour dissemination has been broadly studied at the mRNA level. Several reports show that in the absence of EMT-promoting signals, C3 could enhance metastasis of epithelial cancer cells by loosening cellular junctions^23^, modulating vascularization and endothelial cells function^24^ or promoting survival of cancer cells via increasing their interactions with platelets^25,26^. On the other hand, the ovarian cancer mouse model provided evidence on the correlation between mRNA *C3* expression and lymphatic vasculature^24^. Finally, the complement system also appeared to facilitate early stages of metastasis via modulation of cell adherence in several cancer types^27^.

In breast cancer, local expression of complement inhibitors was reported and perceived as a mechanism of evading immune response and cytocidal complement function. Namely, in primary breast tumours the expression of factor I and CD46 correlated with larger tumour size, lower differentiation index, and poor prognosis^28,29^. Moreover, in animal models of BCa, two groups reported the role of complement in premetastatic niche formation in the lungs^30,31^. Nevertheless, there is little clinical material-derived evidence on the exact role of the complement system in breast cancer dissemination. Still, a recent report by Chatterjee et al.^32^ has comprehensively summarized gene expression changes from healthy to metastatic (sentinel and non-sentinel) lymph node.

In our study, the reduction in local mRNA expression of C3—the central node of all complement activation cascades—in lymph node metastases was confirmed at the protein level. To the best of our knowledge, this is the first evidence for the association between C3 protein expression in breast cancer and metastatic colonization of lymph nodes. By far only one study by Chen and colleagues examined the relationship between the C3 protein and TNM staging and nodal involvement in pancreatic cancer, providing negative results^33^. The report by Vadrevu et al. also demonstrated that in BCa patients the complement proteins, including C3, are produced in both metastatic and metastasis-free nodes, yet the expression level in colonized lymph nodes was substantially higher. Still, the authors conclude that the complement system affects the metastatic process in a context-dependent manner^30^. This is in line with the outcomes of a comprehensive TCGA data analysis by Roumenina et al., investigating the complement system, TME and their prognostic properties in 30 cancer types. Based on survival analysis according to the level of complement-related transcriptome, BCa was classified as a cancer type with uncertain complement significance^18^, perhaps due to the distinct biology of the molecular subtypes of breast tumours. This provides a rationale for further exploration of the complement system role in BCa biology.

One of the limitations of our study was the small size of the exploration group (n = 11) in NanoString analysis. To compensate for that we performed an immunohistochemical validation of selected results on a larger cohort of patients (n = 79), proving the accuracy of our transcriptomic results. Another limitation was the application of NGS (RNA-seq) data on healthy tissues transcriptome for the healthy background normalization step. Since healthy tissue material is rarely included in high-throughput gene expression studies due to both its limited availability and high cost of the analysis, no compatible NanoString data set was available for use in public databases. A growing number of studies proves that NanoString and RNA-seq are compatible for gene expression analysis, both at the single gene and pathway level^34–37^. According to Zhang et al., NanoString and RNA-seq show the highest correlation coefficient among all available transcriptomic methods^38^. Nonetheless, aware of the limitations of comparing data from two different platforms, we did not normalize our NanoString cancer data using the NGS results for healthy tissues; instead, to minimize platform-specific differences, normalization was conducted within the same platform (Fig. 1).

To conclude, based on our findings we propose that the complement system potentially contributes to breast cancer lymphatic spread. We observed reduced mRNA expression of complement system genes in colonized lymph nodes when compared to corresponding primary tumours. Importantly, we also demonstrate that protein expression of complement C3 in cancer cells decreases in the course of metastatic spread and its further decrease in lymph node metastases is linked with patients poor prognosis. Consequently, we hypothesize that in non-metastatic luminal breast cancer patients the local PT expression of complement system-related genes may facilitate their invasion and metastasis, and eventually become decreased to alleviate immune system reaction when the cells reach the lymph node. Due to its potential role in shaping the aggressive phenotype of luminal breast tumours, the complement system appears to be a potential target for cancer treatment and thus merit further studies.

## Methods

### Patients

The study group consisted of 79 non-metastatic luminal BCa patients staged I–III, who underwent surgical treatment at the Medical University Hospital in Gdansk between 2011 and 2013. The study was approved by the Ethical Committee of the Medical University of Gdansk (NKBBN 94/2017) and informed consent was collected from all participants. All experiments were conducted in accordance with the Declaration of Helsinki, REMARK^39^ and STROBE^40^. Patients were characterized by different clinicopathological parameters, including nodal involvement and CTC status, as described previously^41^ and summarised in Table 1. Transcriptome analysis covered archival FFPE samples of PT and 11 LNM from 11 selected N+ patients for whom matched PT-LNM pairs were available. The algorithm of patients/sample selection is depicted in Supplementary Figure S4.

**Table 1:** Clinicopathological characteristics of the cohort.

Parameter	Status	Whole cohort (n = 79)		NanoString group (n = 11)	
		n	%	n	%
Clinical stage	I	22	28	0	0
	II	41	52	6	55
	III	16	20	5	45
T	1	33	42	1	9
	2	41	52	4	36
	3	3	4	6	55
	4	2	3	0	0
N	Negative	36	46	0	0
	Positive	43	54	11	100
CTC	Negative	44	56	6	55
	Epithelial	13	16	0	0
	Mesenchymal	9	11	5	45
	NA	13	16	0	0
Grade	1	14	18	1	9
	2	45	57	4	36
	3	20	25	6	55
Histological type	NST	68	86	11	100
	Other	11	14	0	0
ER status	Negative	4	5	1	9
	Positive	75	95	10	91
PR status	Negative	7	9	0	0
	Positive	72	91	11	100
HER2 status	Negative	57	72	8	73
	Positive	22	28	3	27
Molecular subtype	lumA	31	39	2	18
	lumB HER2-	26	33	6	55
	lumB HER2+	22	28	3	27

### nCounter transcriptome profiling of primary breast cancer and corresponding lymph node metastasis fragments

Total RNA was isolated from archival FFPE blocks using RNeasy Mini Kit (Qiagen) and the expression of 730 target genes was evaluated with nCounter PanCancer Immune Profiling Panel (NanoString Technologies), as reported previously^42^.

For each analysed sample, background correction and normalization against global mean were performed as described^42^, using nSolver 4.0 software (NanoString Technologies). PT and LNM samples were normalized together. In brief, the background level was estimated by thresholding over the mean plus 2 standard deviations of the negative control counts. Subsequently, the data were normalized according to the global mean of the counts of positive controls and 4 most stably expressed housekeeping genes—*ABCF1*, *EDC3*, *HDAC3*, and *CNOT4*. The negative and positive control probes were included in the assay. Following normalization, low-expression genes (log2 mean count in all samples < 6) were excluded, leaving 593 target genes for analysis.

### Healthy tissues transcriptome profiles from GeneCards database

Illumina Body Map expression data for normal lymph node (LYMPH NODE) and normal breast tissue (BREAST) generated within the Genotype-Tissue Expression (GTEx) project^8^ and deposited in the GeneCards database^43^ were obtained as a courtesy of Weizmann Institute of Science. 578 out of 593 genes expressed in the NanoString data were present in the Illumina Body Map dataset. Low-expression genes (log2 FPKM in each normal tissues < 5) were excluded, leaving 360 target genes for analysis.

### Transcriptome data analysis

Analysis was performed for 360 genes expressed in both the NanoString and Illumina Body Map datasets (as depicted in Fig. 1, genes listed in Supplementary Table S3). The NanoString dataset comprised 11 matched LNM-PT pairs of BCa samples, whereas the Illumina Body Map dataset included the healthy control tissues—LYMPH NODE and BREAST. In brief, all data were log2 transformed and a log2 LYMPH NODE/BREAST (HEALTHY) ratio was calculated for each gene using Illumina Body Map data. In parallel, a matched log2 LNM/PT (CANCER) ratio was calculated for each patient using NanoString data. Subsequently, the HEALTHY ratio was subtracted from the matched CANCER ratio, giving a healthy background-normalized log2 LNM/PT ratio (further referred to as normalized LNM/PT ratio) for each patient. Eventually, the median normalized LNM/PT ratio was calculated for each gene based on the data of the whole cohort.

Genes with median normalized LNM/PT ratio > 1 were considered LNM-enriched compared to healthy lymph node (Supplementary Figure S5A). Genes with median normalized LNM/PT ratio < −1 were considered LNM-depleted compared to healthy lymph node (Supplementary Figure S5B). LNM-enriched/depleted genes were then analysed for changes in gene expression level from PT to LNM in reference to the healthy background. Genes with LYMPH NODE < BREAST and median normalized LNM > PT expression tendencies were considered LNM-upregulated in comparison to PT (Supplementary Figure S5C), while genes with LYMPH NODE > BREAST and median normalized LNM < PT expression tendencies were considered LNM-downregulated in comparison to PT (Supplementary Figure S5D).

### Immunohistochemical evaluation of complement C3 protein

Tissue microarrays (TMA) comprising five 1-mm diameter tumour samples per each patient were prepared as previously described^44^. In brief, to detect C3, TMA sections were deparaffinised and treated with citrate buffer (pH 6, Dako) for 10 min and Peroxidase-Blocking Solution (Dako) for 5 min. The sections were incubated for 1 h at RT with polyclonal rabbit anti-C3 antibody (NBP1-32080, NOVUS Biologicals) diluted 1:250, envisioned by EnVision Kit, Rabbit/Mouse (Dako) and counterstained with haematoxylin (Sigma Aldrich). All tumour samples were evaluated in both tumour cells and surrounding stroma. Intensity of the staining and its semi-quantitative presence was documented. Staining was categorized based on the intensity as negative (i.e. no or weak expression), or positive (i.e. moderate to strong expression), and the tumour samples were scored for stromal, tumoral and overall (i.e. tumour cells and stroma) C3 expression. For each specimen, maximum record out of all examined and informative tumour samples was assigned for further analysis.

### Statistical analysis

Data were analysed and visualized using R computing environment (3.6.1)^45^ and GraphPad Prism 8 (GraphPad Software) licensed for Medical University of Gdańsk. Differences in the distribution of C3 expression status were estimated with Fisher’s exact test. Association between tumoral C3 and overall survival was evaluated using log-rank test. Statistical significance was inferred for *p* values ≤ 0.05.

Selected genes were functionally annotated with Reactome pathways using over-representation analysis tool by ConsensusPathDB^46^. Interactions between protein products of selected genes were visualized using STRING v11^47^.

## Supplementary Information

Supplementary Legends.

Supplementary Figure S1.

Supplementary Figure S2.

Supplementary Figure S3.

Supplementary Figure S4.

Supplementary Figure S5.

Supplementary Table S1.

Supplementary Table S2.

Supplementary Table S3.

## Data Availability

The datasets generated and/or analysed during the current study are available from the corresponding author on request.
