# Supplementary material for: Reduced expression of innate immunity-related genes in lymph node metastases of luminal breast cancer patients
Source: Sci Rep. 2021 Mar 3;11:5097. doi: 10.1038/s41598-021-84568-0 (PMC7930267; doi:10.1038/s41598-021-84568-0)
Supplement: Supplementary file 1 — Supplementary Legends. [file 41598_2021_84568_MOESM1_ESM.pdf]

SUPPLEMENTARY INFORMATION

Reduced expression of innate immunity-related genes in lymph node metastases of luminal breast cancer patients

Marta Popeda[1], Aleksandra Markiewicz[1], Tomasz Stokowy[2], Jolanta Szade[3], Magdalena Niemira[4], Adam Kretowski[4], Natalia Bednarz-Knoll[1], Anna J. Zaczek[1,*]

[1]Laboratory of Translational Oncology, Intercollegiate Faculty of Biotechnology, Medical University of Gdansk, 80-211 Gdansk, Poland

[2]Department of Clinical Science, University of Bergen, 5021 Bergen, Norway

[3]Department of Pathomorphology, Medical University of Gdansk, 80-211 Gdansk, Poland

[4]Clinical Research Centre, Medical University of Bialystok, 15-276 Bialystok, Poland

*correspondence: azaczek@gumed.edu.pl (AJZ); tel.: +48-58-349-14-38

Supplementary Figure S1. Expression of LNM-up/downregulated genes in BCa and healthy tissues

Left plot depicts expression of a given gene in cancer (PT and LNM – pink points) and healthy (BREAST and LYMPHNODE – green points) tissues; lines connect corresponding PT-LNM tissues. Middle plot illustrates distribution of matched CANCER ratios (pink box) and HEALTHY ratio (green diamond) calculated for a given gene; grey dashed line represents the cut-off for direction analysis (equal 0). Right plot illustrates distribution of normalized LNM/PT ratios (grey box) calculated by subtracting HEALTHY ratio from each matched CANCER ratio; median normalized LNM/PT ratio of the whole cohort is provided above the plot; grey dashed lines represent the cut-off for LNM-enrichment (equal 1) and LNM-depletion (equal -1).

Supplementary Figure S2. Concordance between *C3* mRNA and tumoral C3 protein expression

mRNA and protein levels of C3 compared in NanoString group (PT and LNM tissue samples analysed together); mRNA expression classified as low/high according to median; distribution estimated with Fisher exact test.

Supplementary Figure S3. Loss of tumoral C3 in LNM is a negative prognostic factor in luminal BCa

Difference estimated with log-rank test; OS – overall survival.

Supplementary Figure S4. Scheme of patients and/or samples selection

Supplementary Figure S5. Detailed flow of data analysis exemplified for LNM-enriched, -depleted, -upregulated and -downregulated genes

(A) LNM-enriched (i.e. LNM/PT ratio >1), (B) LNM-depleted (i.e. LNM/PT ratio <-1), (C) LNM-upregulated (i.e. LNM>PT & LYMPH NODE<BREAST) and (D) LNM-downregulated gene (i.e. LNM<PT & LYMPH NODE>BREAST). Left plot depicts expression of a given gene in cancer (PT and LNM – pink points) and healthy (BREAST and LYMPHNODE – green points) tissues; lines connect corresponding PT-LNM tissues. Middle plot illustrates distribution of matched CANCER ratios (pink box) and HEALTHY ratio (green diamond) calculated for a given gene; grey dashed line represents the cut-off for direction analysis (equal 0). Right plot illustrates distribution of normalized LNM/PT ratios (grey box) calculated by subtracting HEALTHY ratio from each matched CANCER ratio; median normalized LNM/PT ratio of the whole cohort is provided above the plot; grey dashed lines represent the cut-off for LNM-enrichment (equal 1) and LNM-depletion (equal -1).

Supplementary Table S1. List of LNM-enriched, -depleted, -upregulated and -downregulated genes

Supplementary Table S2. List of Reactome pathways enriched in LNM-downregulated genes

Supplementary Table S3. List of analysed genes
