# Supplementary material for: Reduced expression of innate immunity-related genes in lymph node metastases of luminal breast cancer patients
Source: Sci Rep. 2021 Mar 3;11:5097. doi: 10.1038/s41598-021-84568-0 (PMC7930267; doi:10.1038/s41598-021-84568-0)
Supplement: Supplementary file 2 — Supplementary Figure S1. [file 41598_2021_84568_MOESM2_ESM.pdf]

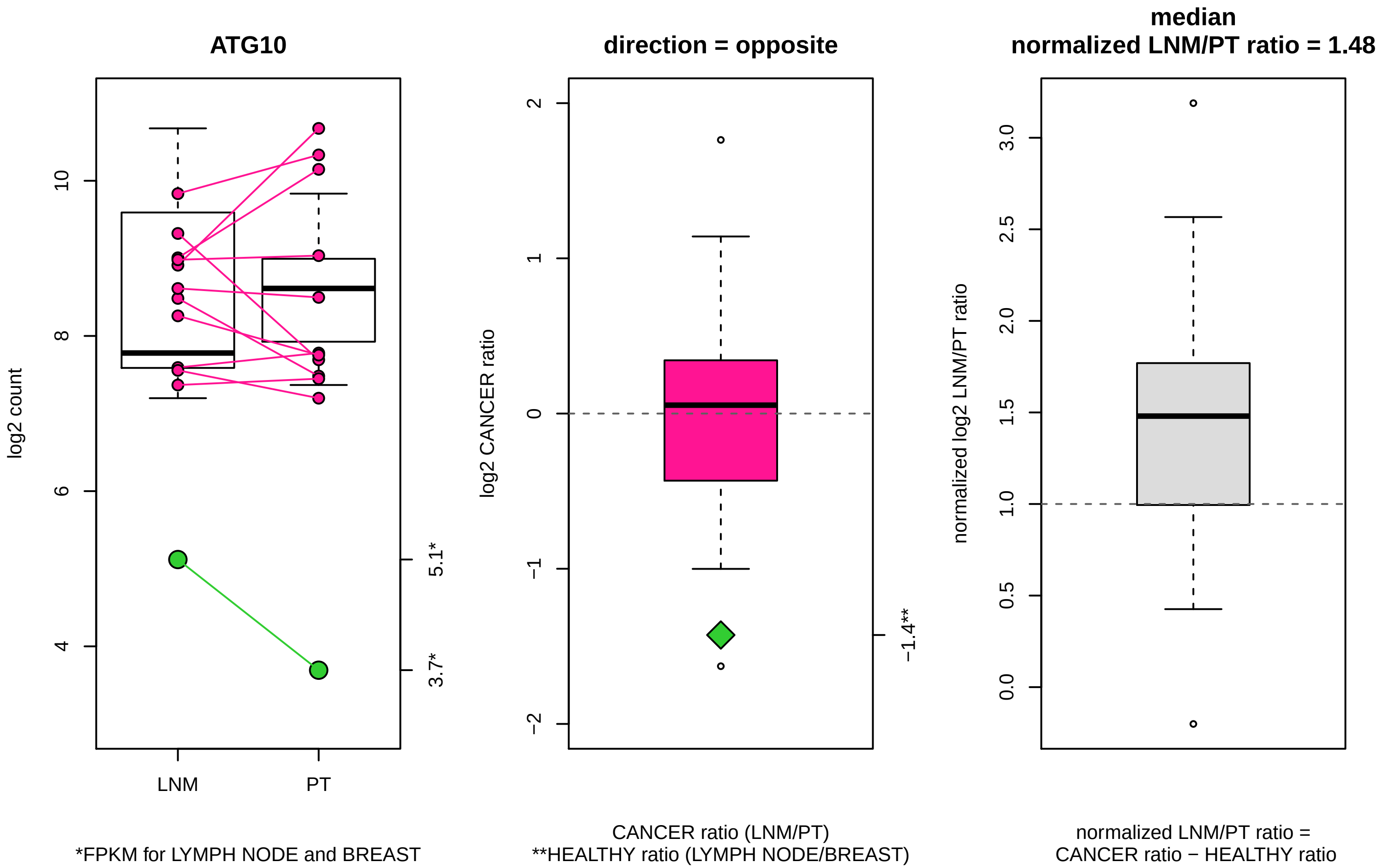
ATG10
log2 count
10
8
6
4
LNM
PT
5.1*
3.7*
*FPKM for LYMPH NODE and BREAST
direction = opposite
log2 CANCER ratio
2
1
0
−1
−2
−1.4**
CANCER ratio (LNM/PT)
**HEALTHY ratio (LYMPH NODE/BREAST)
median
normalized LNM/PT ratio = 1.48
normalized log2 LNM/PT ratio
3.0
2.5
2.0
1.5
1.0
0.5
0.0
normalized LNM/PT ratio =
CANCER ratio − HEALTHY ratio

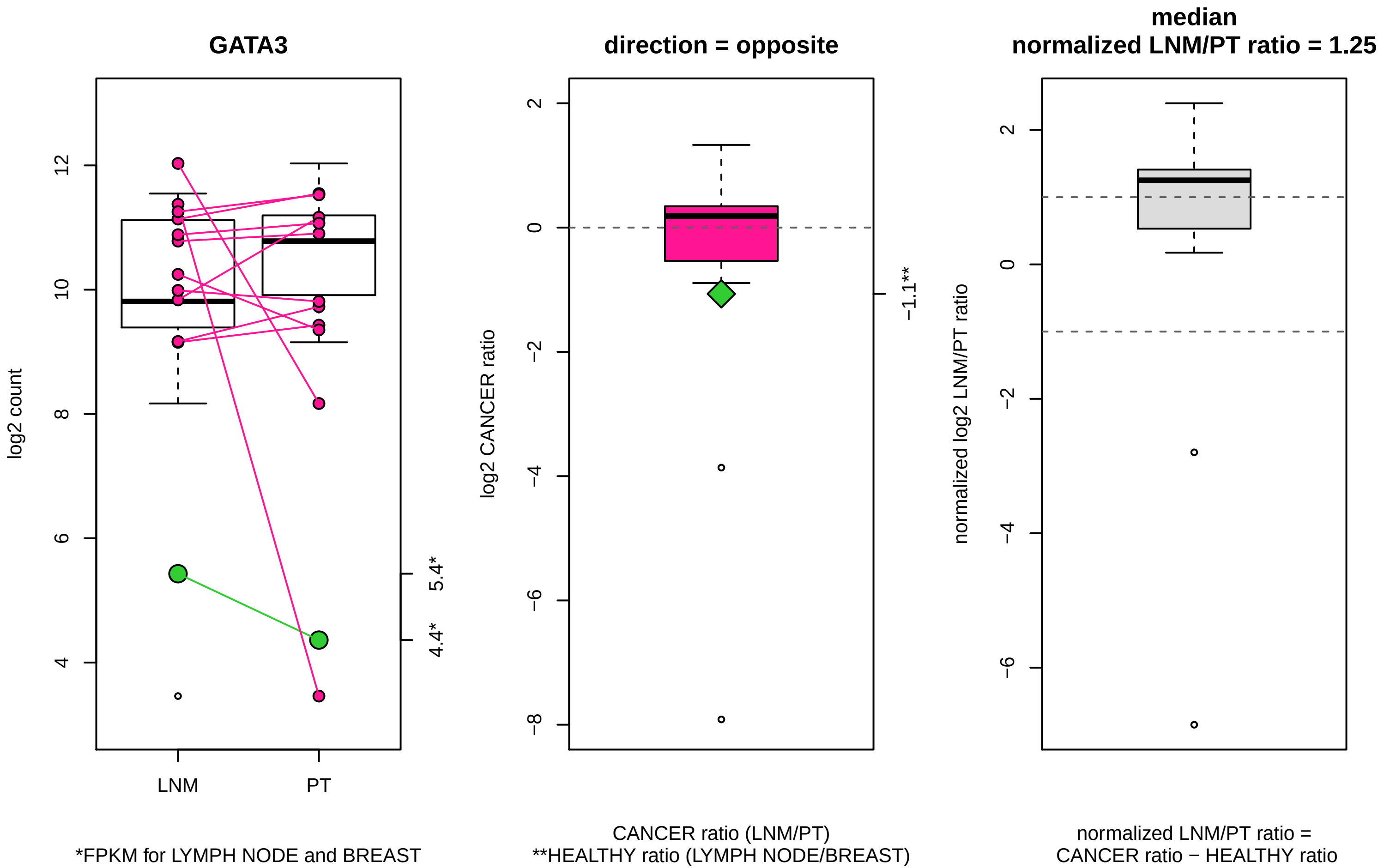

GATA3
direction = opposite
median
normalized LNM/PT ratio = 1.25
log2 count
LNM
PT
5.4*
4.4*
log2 CANCER ratio
−1.1**
normalized log2 LNM/PT ratio
*FPKM for LYMPH NODE and BREAST
CANCER ratio (LNM/PT)
**HEALTHY ratio (LYMPH NODE/BREAST)
normalized LNM/PT ratio =
CANCER ratio − HEALTHY ratio

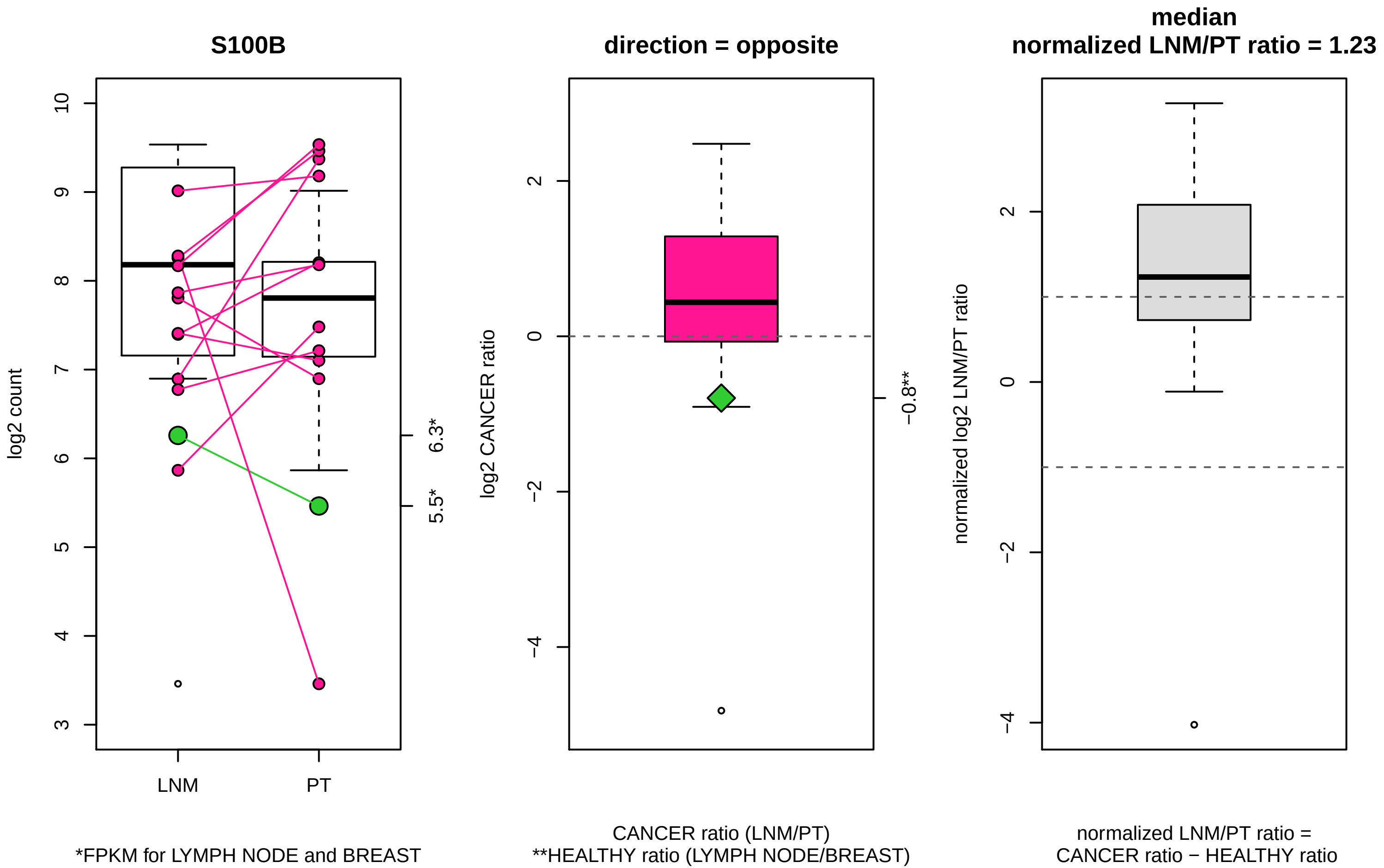
S100B
direction = opposite
median
normalized LNM/PT ratio = 1.23
log2 count
LNM
PT
6.3*
5.5*
log2 CANCER ratio
−0.8**
normalized log2 LNM/PT ratio
*FPKM for LYMPH NODE and BREAST
CANCER ratio (LNM/PT)
**HEALTHY ratio (LYMPH NODE/BREAST)
normalized LNM/PT ratio =
CANCER ratio − HEALTHY ratio

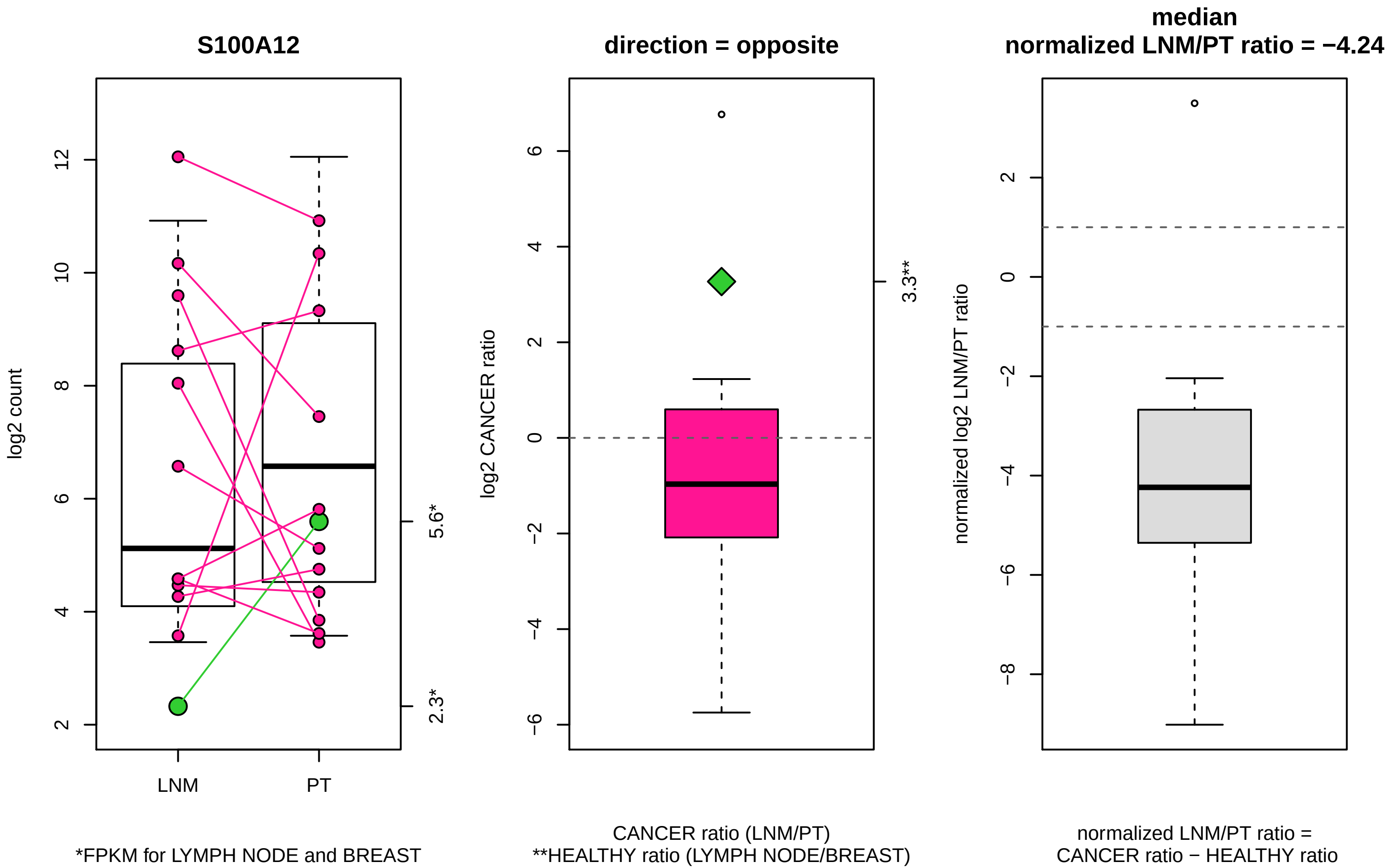

S100A12
direction = opposite
median
normalized LNM/PT ratio = −4.24
log2 count
LNM
PT
5.6*
2.3*
log2 CANCER ratio
3.3**
normalized log2 LNM/PT ratio
*FPKM for LYMPH NODE and BREAST
CANCER ratio (LNM/PT)
**HEALTHY ratio (LYMPH NODE/BREAST)
normalized LNM/PT ratio =
CANCER ratio − HEALTHY ratio

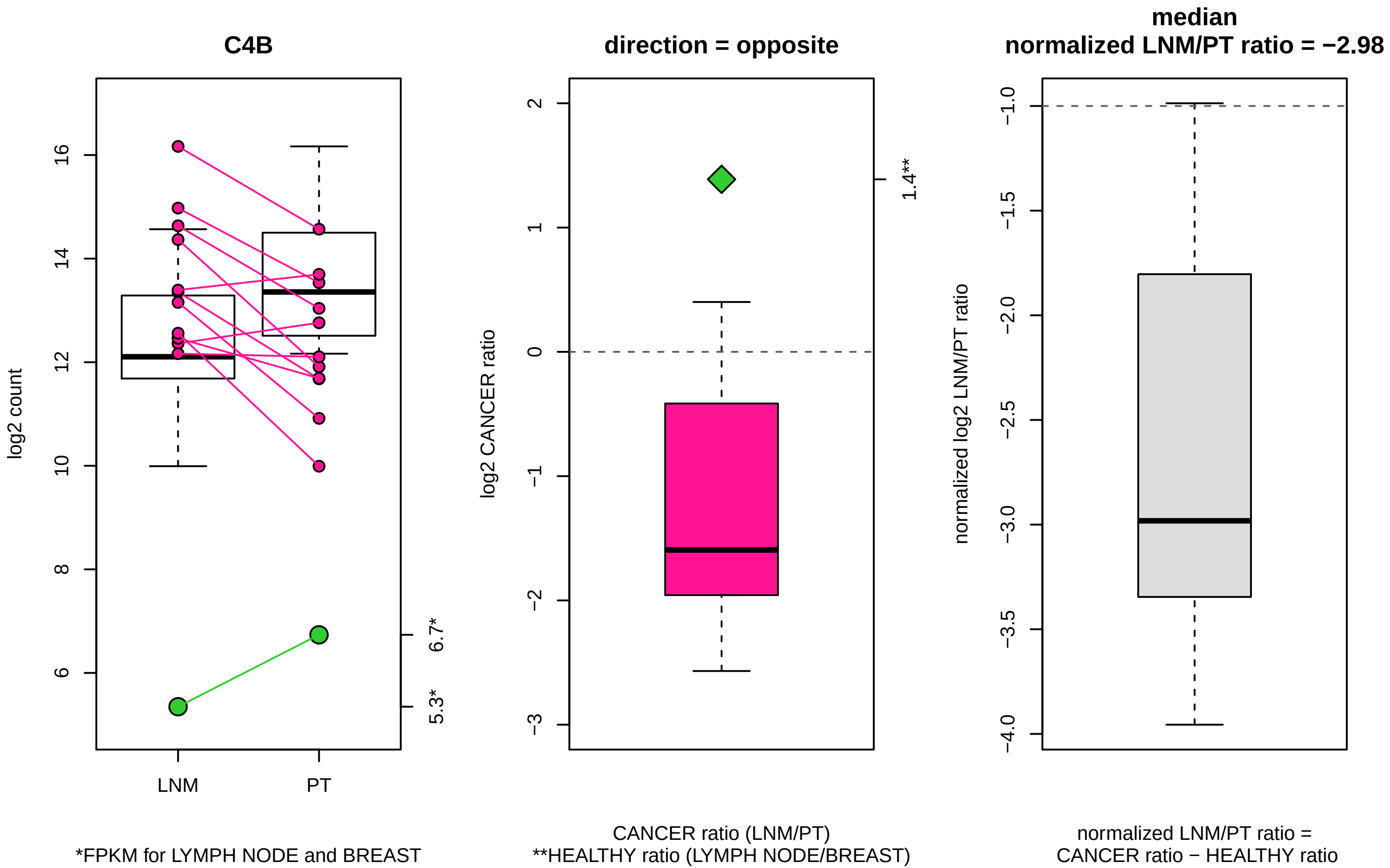
C4B
direction = opposite
median
normalized LNM/PT ratio = −2.98
log2 count
16
14
12
10
8
6
6.7*
5.3*
LNM
PT
*FPKM for LYMPH NODE and BREAST
log2 CANCER ratio
2
1
0
−1
−2
−3
1.4**
CANCER ratio (LNM/PT)
**HEALTHY ratio (LYMPH NODE/BREAST)
normalized log2 LNM/PT ratio
−1.0
−1.5
−2.0
−2.5
−3.0
−3.5
−4.0
normalized LNM/PT ratio =
CANCER ratio − HEALTHY ratio

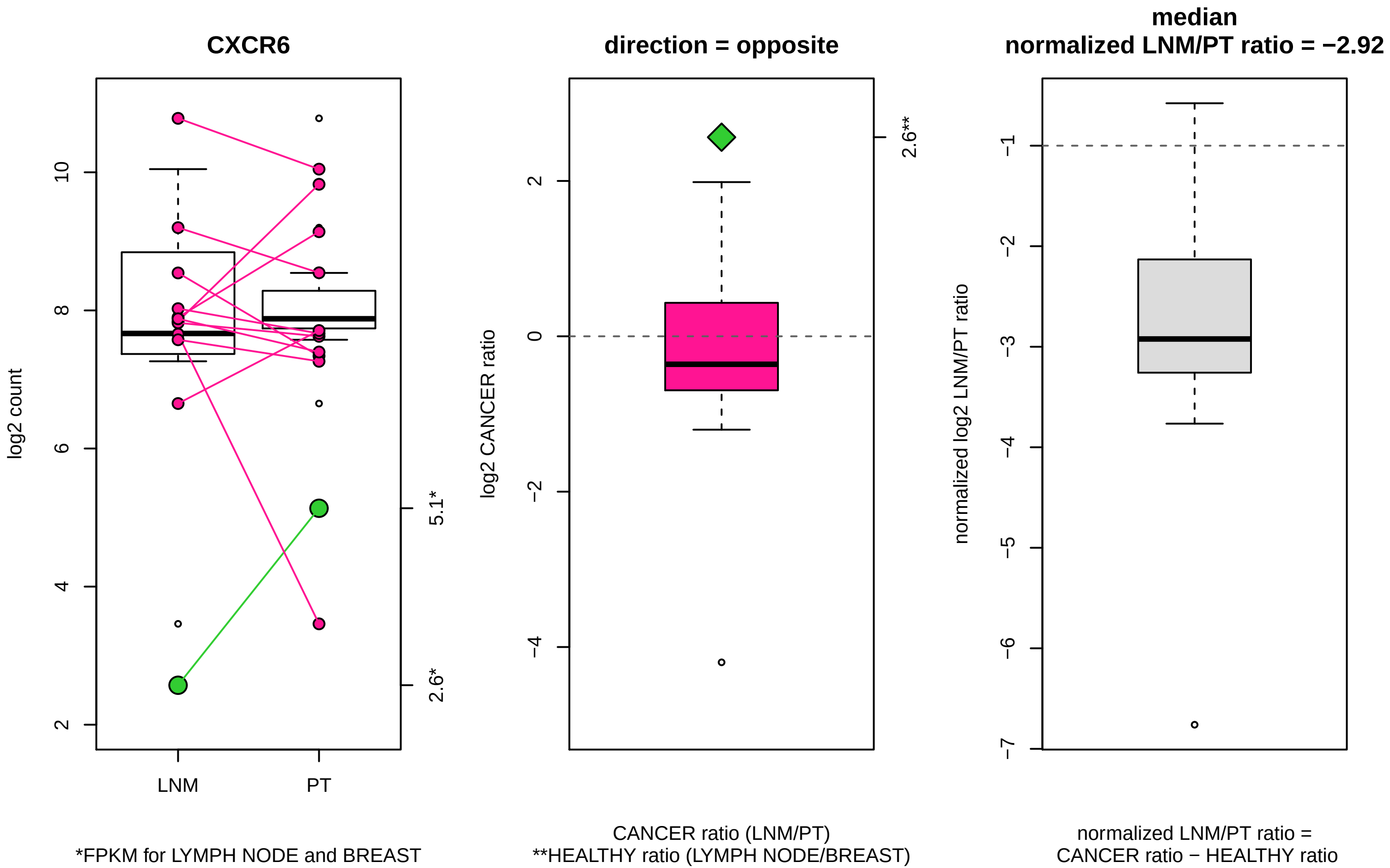
CXCR6
direction = opposite
median
normalized LNM/PT ratio = −2.92
log2 count
LNM
PT
5.1*
2.6*
log2 CANCER ratio
2.6**
normalized log2 LNM/PT ratio
*FPKM for LYMPH NODE and BREAST
CANCER ratio (LNM/PT)
**HEALTHY ratio (LYMPH NODE/BREAST)
normalized LNM/PT ratio =
CANCER ratio − HEALTHY ratio

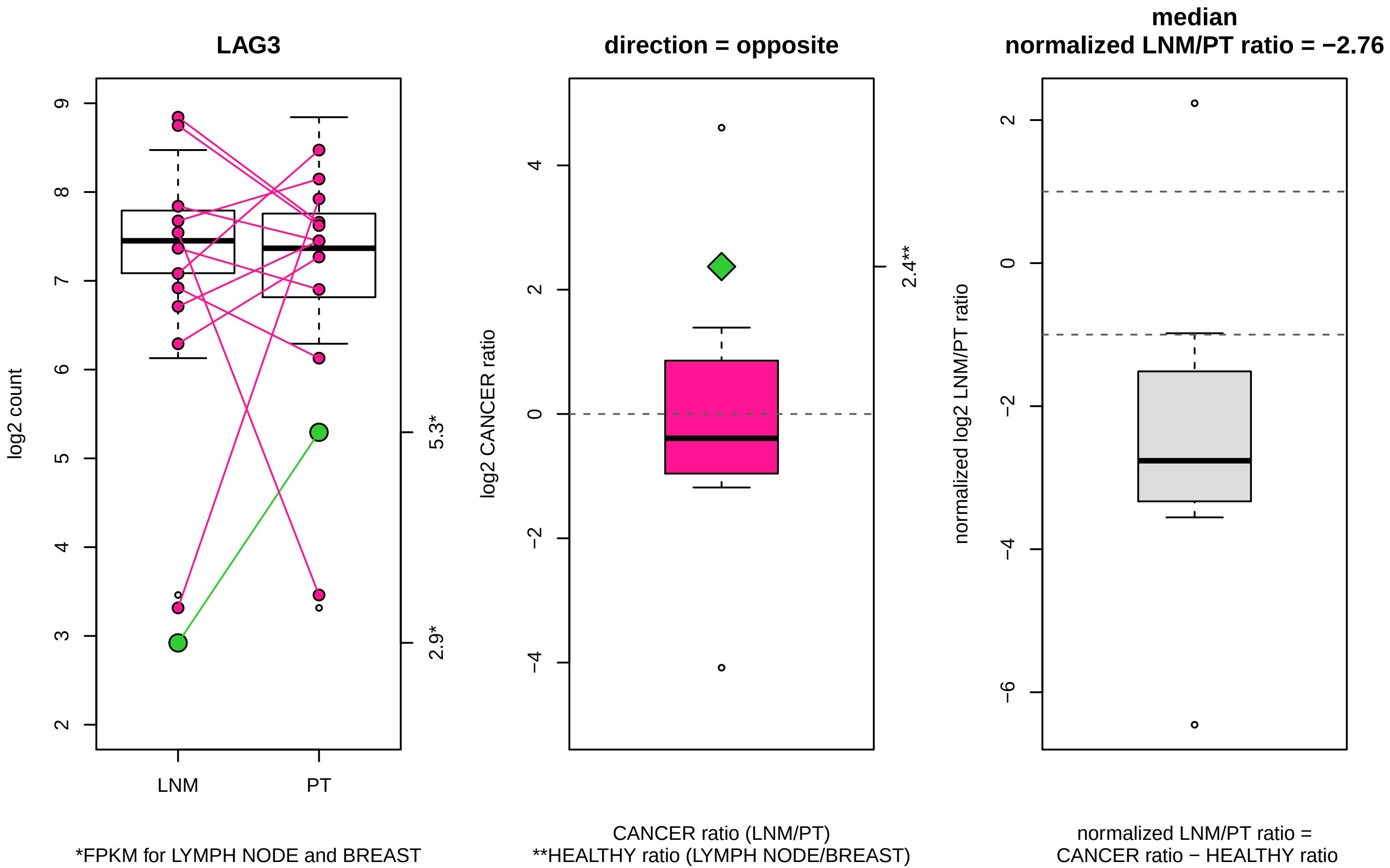
LAG3
log2 count
9
8
7
6
5
4
3
2
LNM
PT
5.3*
2.9*
*FPKM for LYMPH NODE and BREAST
direction = opposite
log2 CANCER ratio
4
2
0
−2
−4
2.4**
CANCER ratio (LNM/PT)
**HEALTHY ratio (LYMPH NODE/BREAST)
median
normalized LNM/PT ratio = −2.76
normalized log2 LNM/PT ratio
2
0
−2
−4
−6
normalized LNM/PT ratio =
CANCER ratio − HEALTHY ratio

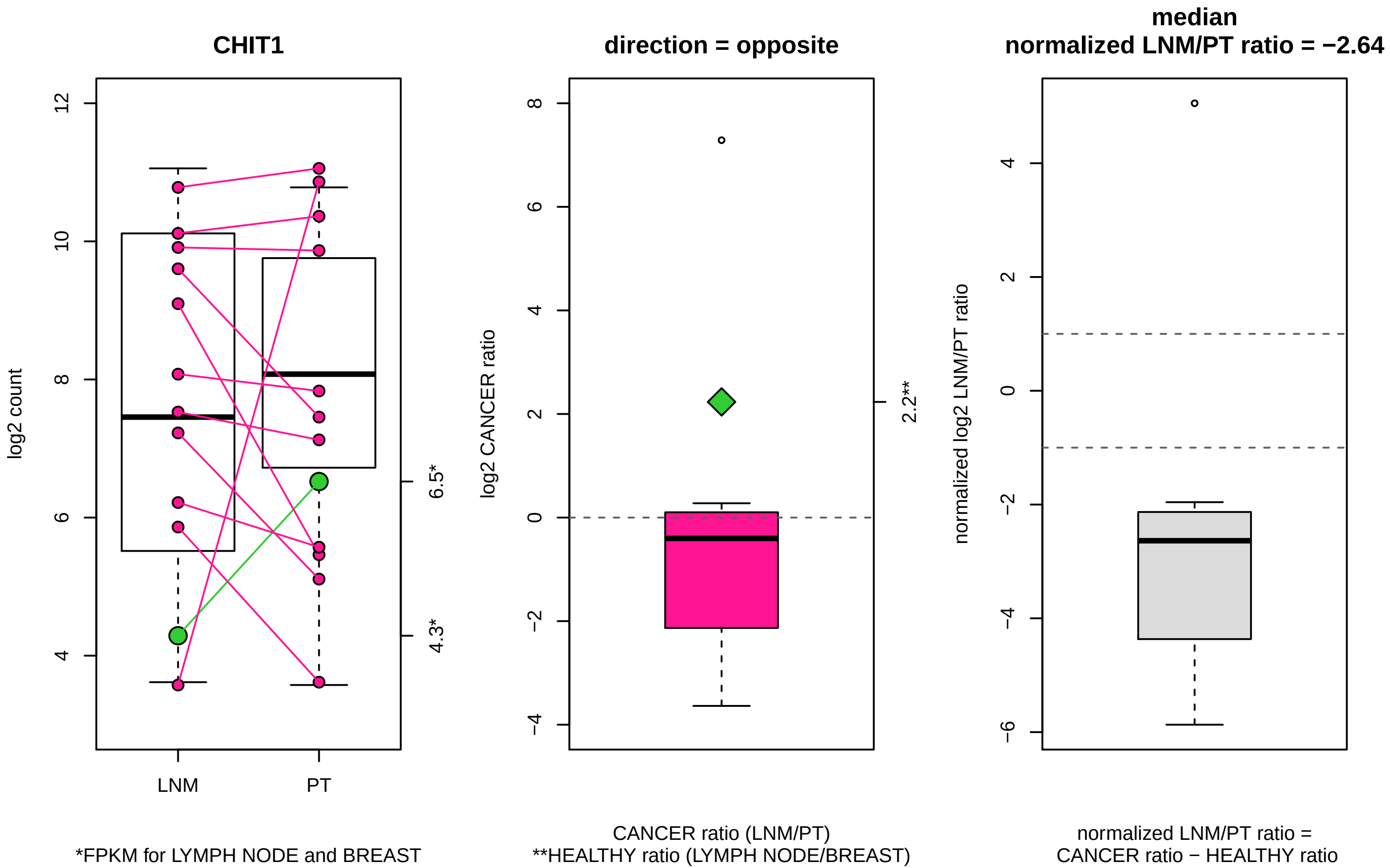

CHIT1
direction = opposite
median
normalized LNM/PT ratio = −2.64
log2 count
12
10
8
6
4
LNM
PT
6.5*
4.3*
log2 CANCER ratio
8
6
4
2
0
−2
−4
2.2**
normalized log2 LNM/PT ratio
4
2
0
−2
−4
−6
*FPKM for LYMPH NODE and BREAST
CANCER ratio (LNM/PT)
**HEALTHY ratio (LYMPH NODE/BREAST)
normalized LNM/PT ratio =
CANCER ratio − HEALTHY ratio

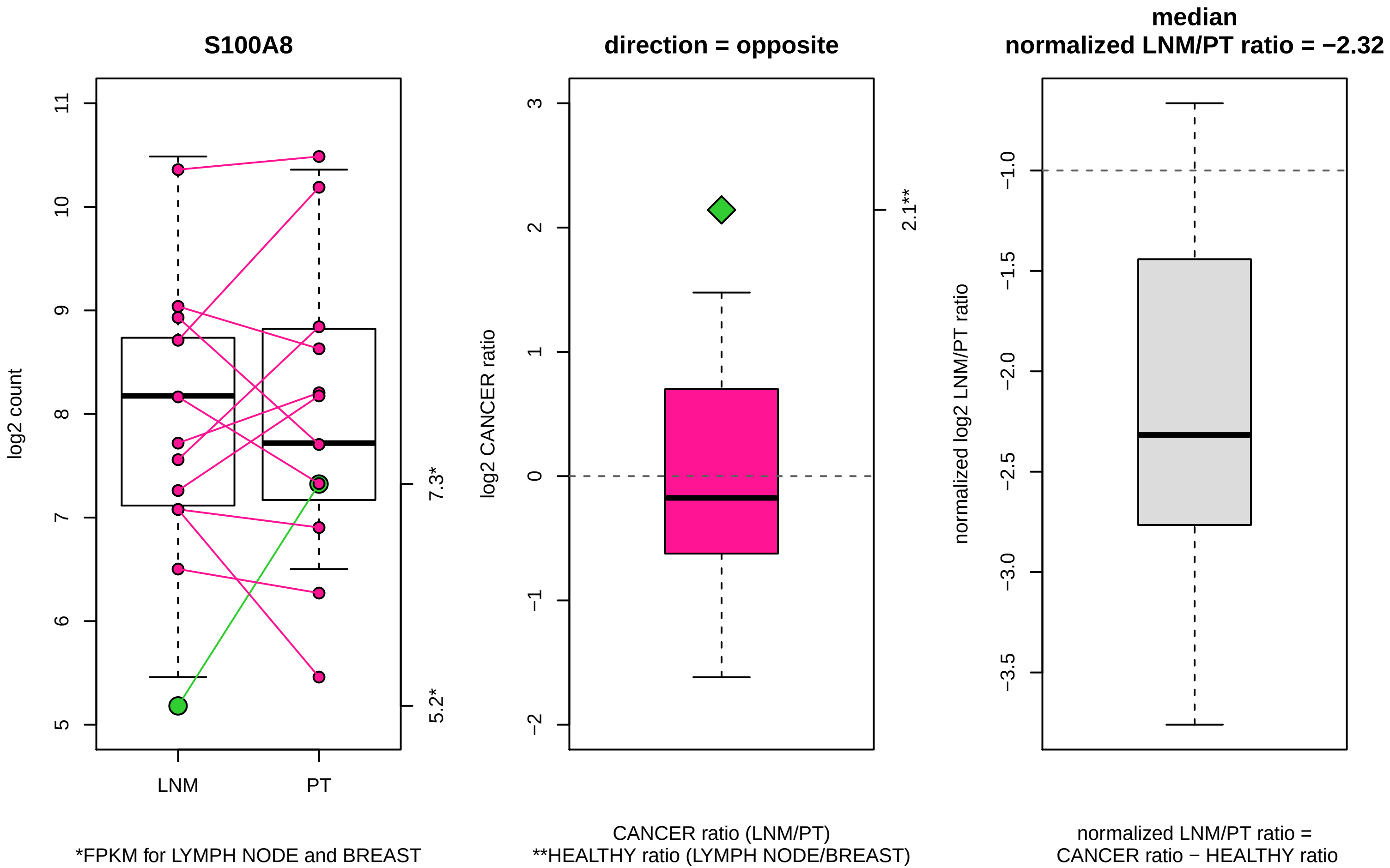

S100A8
log2 count
11
10
9
8
7
6
5
7.3*
5.2*
LNM
PT
*FPKM for LYMPH NODE and BREAST
direction = opposite
log2 CANCER ratio
3
2
1
0
−1
−2
2.1**
CANCER ratio (LNM/PT)
**HEALTHY ratio (LYMPH NODE/BREAST)
median
normalized LNM/PT ratio = −2.32
normalized log2 LNM/PT ratio
−1.0
−1.5
−2.0
−2.5
−3.0
−3.5
normalized LNM/PT ratio =
CANCER ratio − HEALTHY ratio

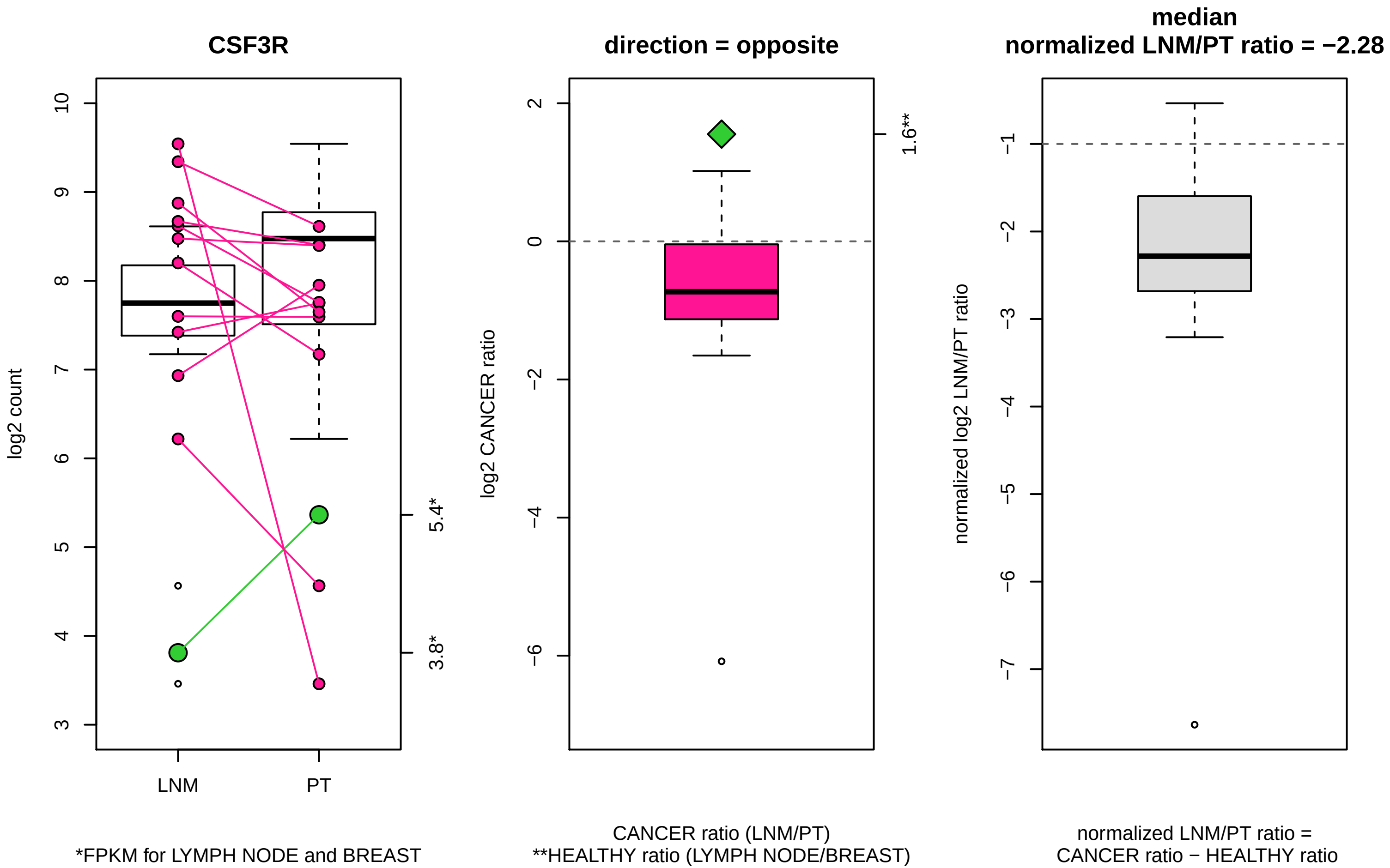

CSF3R
direction = opposite
median
normalized LNM/PT ratio = −2.28
log2 count
LNM
PT
5.4*
3.8*
log2 CANCER ratio
1.6**
normalized log2 LNM/PT ratio
*FPKM for LYMPH NODE and BREAST
CANCER ratio (LNM/PT)
**HEALTHY ratio (LYMPH NODE/BREAST)
normalized LNM/PT ratio =
CANCER ratio − HEALTHY ratio

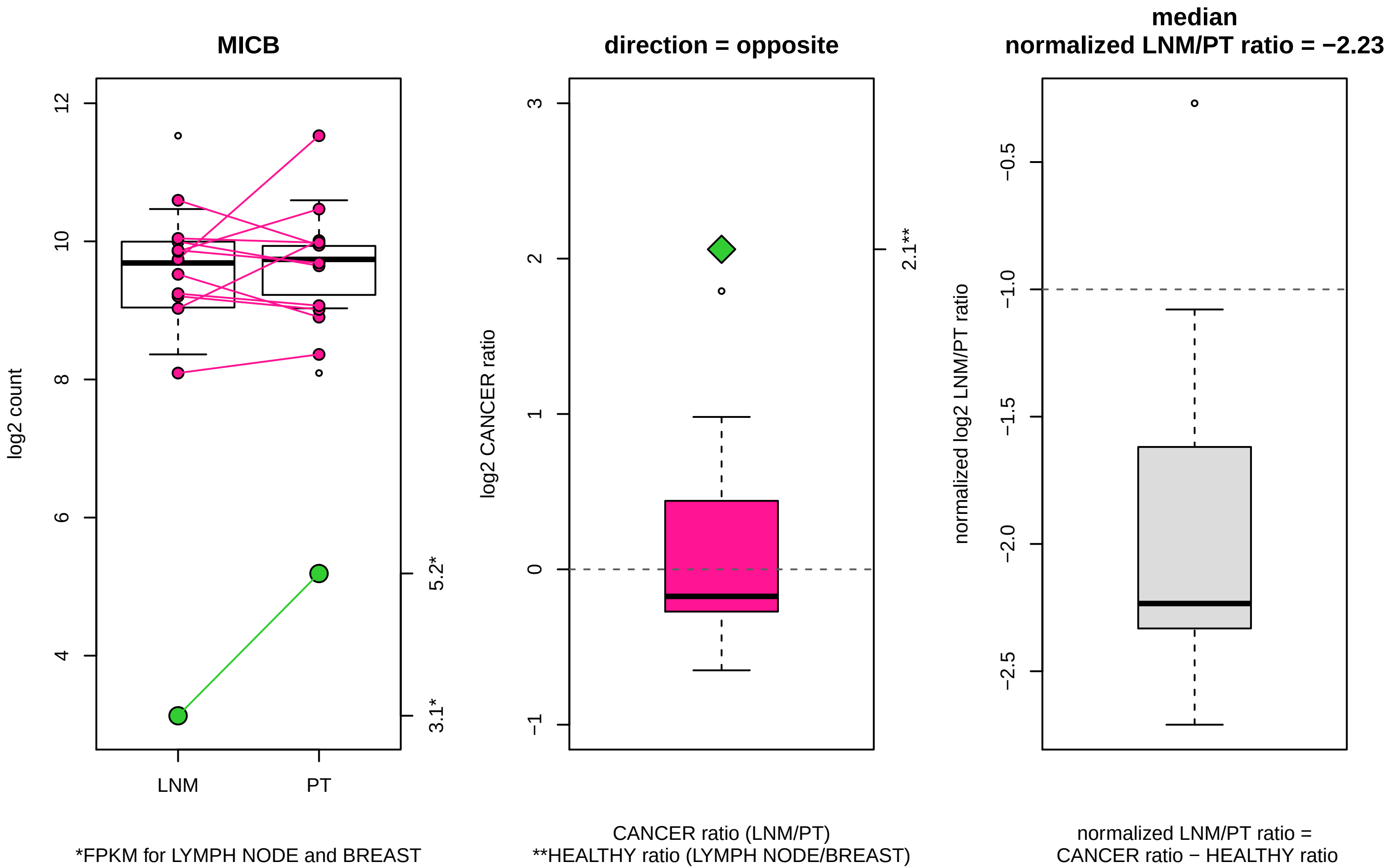

MICB
log2 count
LNM
PT
5.2*
3.1*
*FPKM for LYMPH NODE and BREAST
direction = opposite
log2 CANCER ratio
2.1**
CANCER ratio (LNM/PT)
**HEALTHY ratio (LYMPH NODE/BREAST)
median
normalized LNM/PT ratio = −2.23
normalized log2 LNM/PT ratio
normalized LNM/PT ratio =
CANCER ratio − HEALTHY ratio

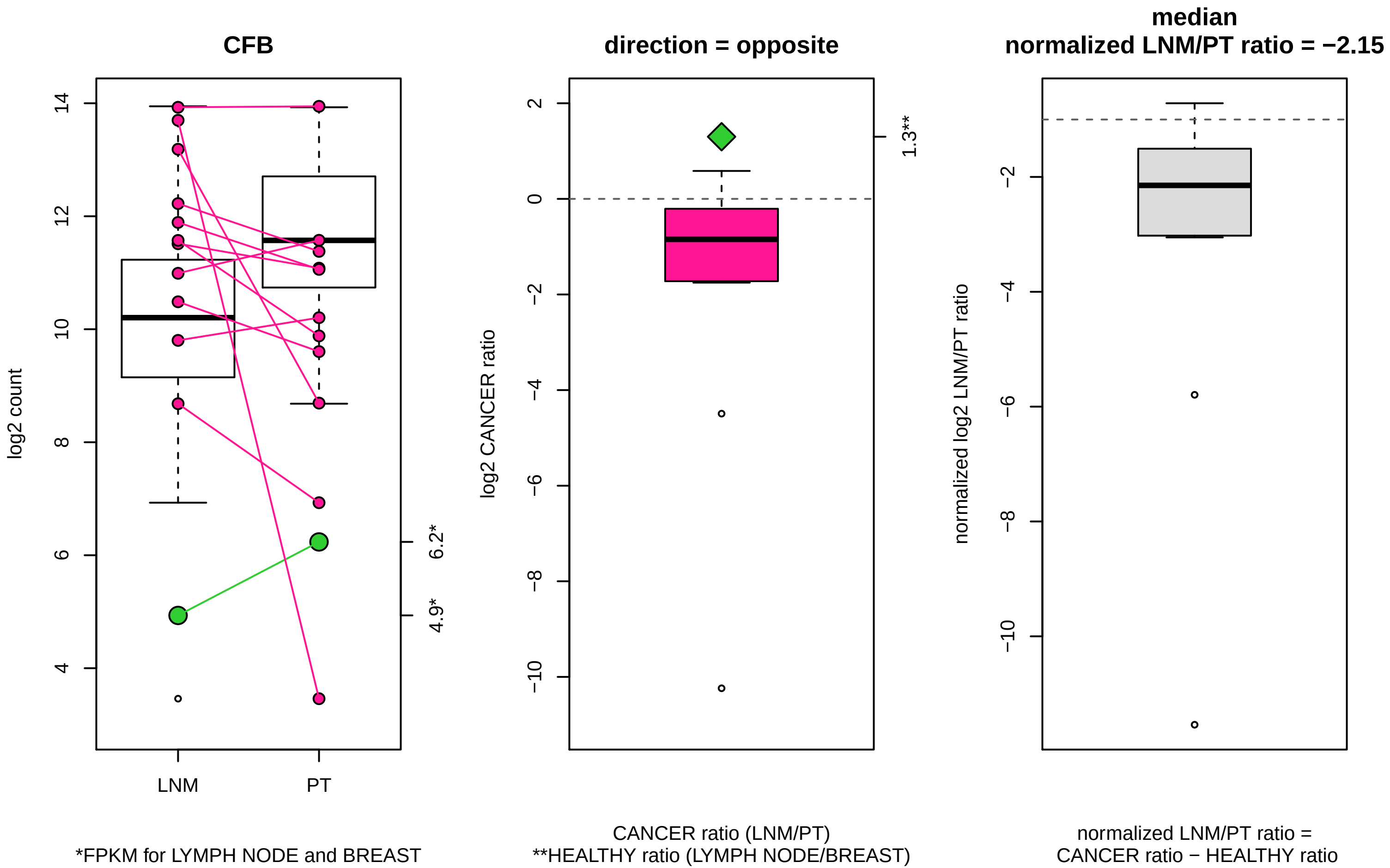

CFB
log2 count
14
12
10
8
6
4
LNM
PT
6.2*
4.9*
*FPKM for LYMPH NODE and BREAST
direction = opposite
log2 CANCER ratio
2
0
−2
−4
−6
−8
−10
1.3**
CANCER ratio (LNM/PT)
**HEALTHY ratio (LYMPH NODE/BREAST)
median
normalized LNM/PT ratio = −2.15
normalized log2 LNM/PT ratio
−2
−4
−6
−8
−10
normalized LNM/PT ratio =
CANCER ratio − HEALTHY ratio

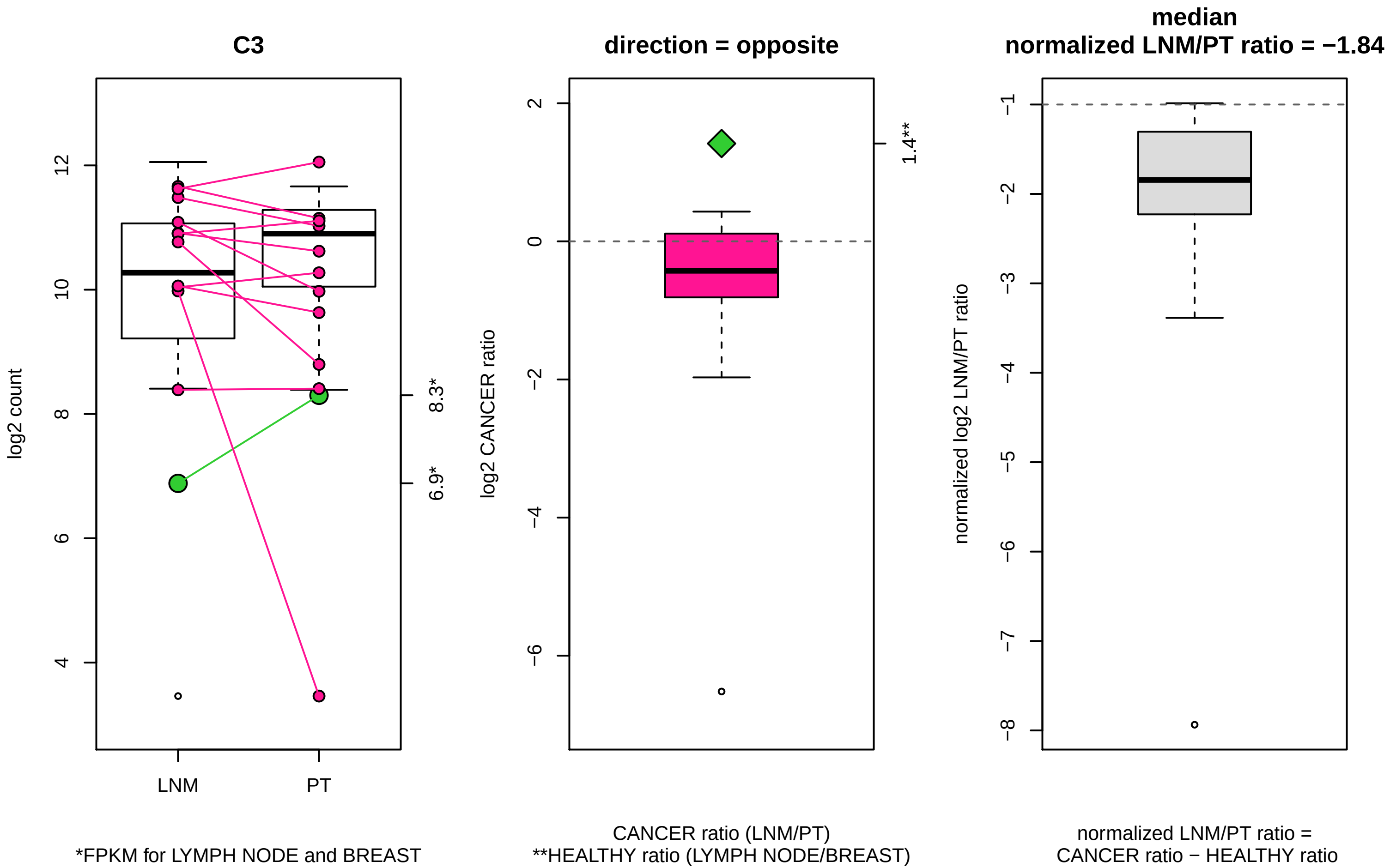
C3
log2 count
12
10
8
6
4
LNM
PT
8.3*
6.9*
*FPKM for LYMPH NODE and BREAST
direction = opposite
log2 CANCER ratio
2
0
−2
−4
−6
1.4**
CANCER ratio (LNM/PT)
**HEALTHY ratio (LYMPH NODE/BREAST)
median
normalized LNM/PT ratio = −1.84
normalized log2 LNM/PT ratio
−1
−2
−3
−4
−5
−6
−7
−8
normalized LNM/PT ratio =
CANCER ratio − HEALTHY ratio

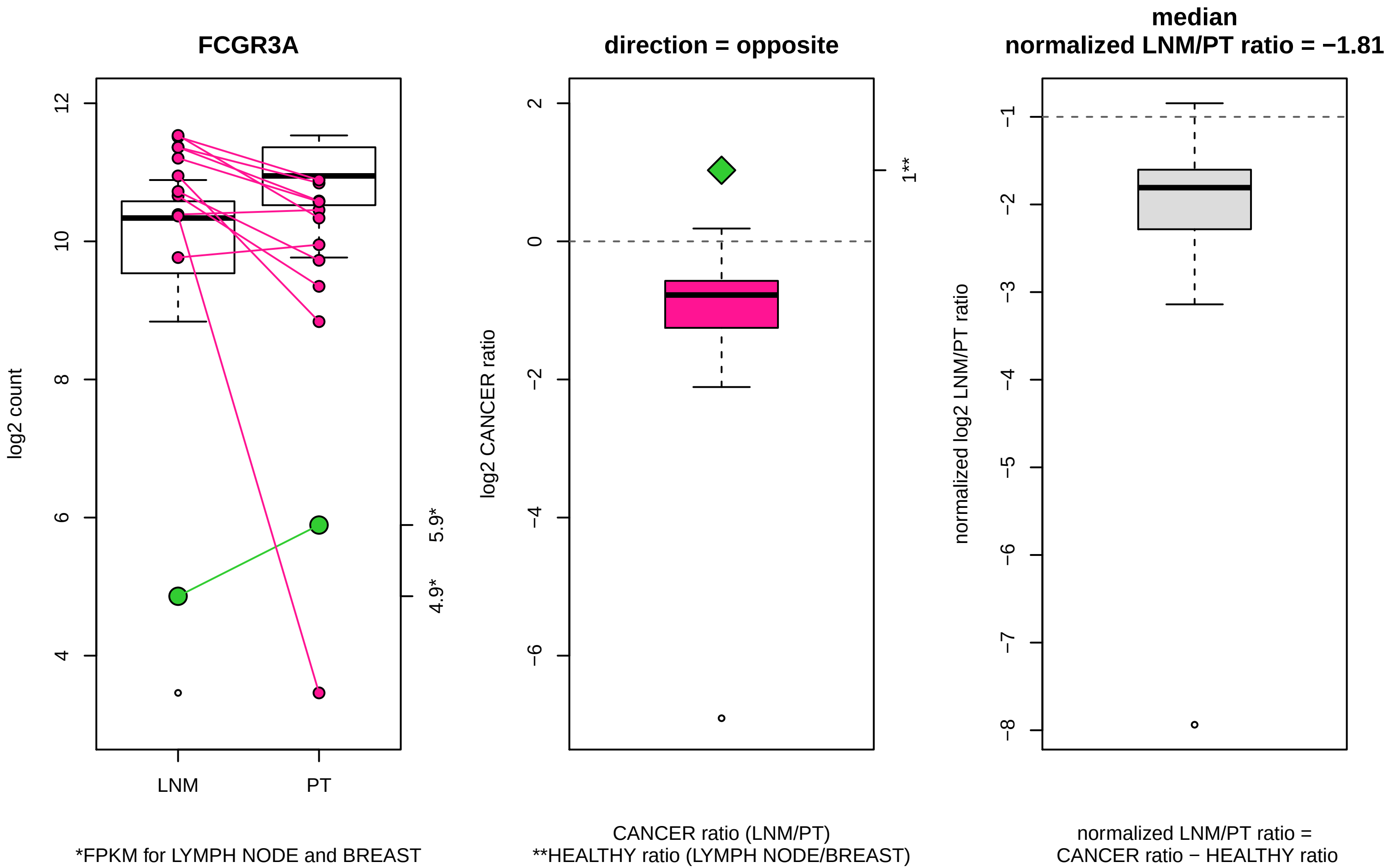
FCGR3A
direction = opposite
median
normalized LNM/PT ratio = −1.81
log2 count
12
10
8
6
4
5.9*
4.9*
LNM
PT
*FPKM for LYMPH NODE and BREAST
log2 CANCER ratio
2
0
−2
−4
−6
1**
CANCER ratio (LNM/PT)
**HEALTHY ratio (LYMPH NODE/BREAST)
normalized log2 LNM/PT ratio
−1
−2
−3
−4
−5
−6
−7
−8
normalized LNM/PT ratio =
CANCER ratio − HEALTHY ratio

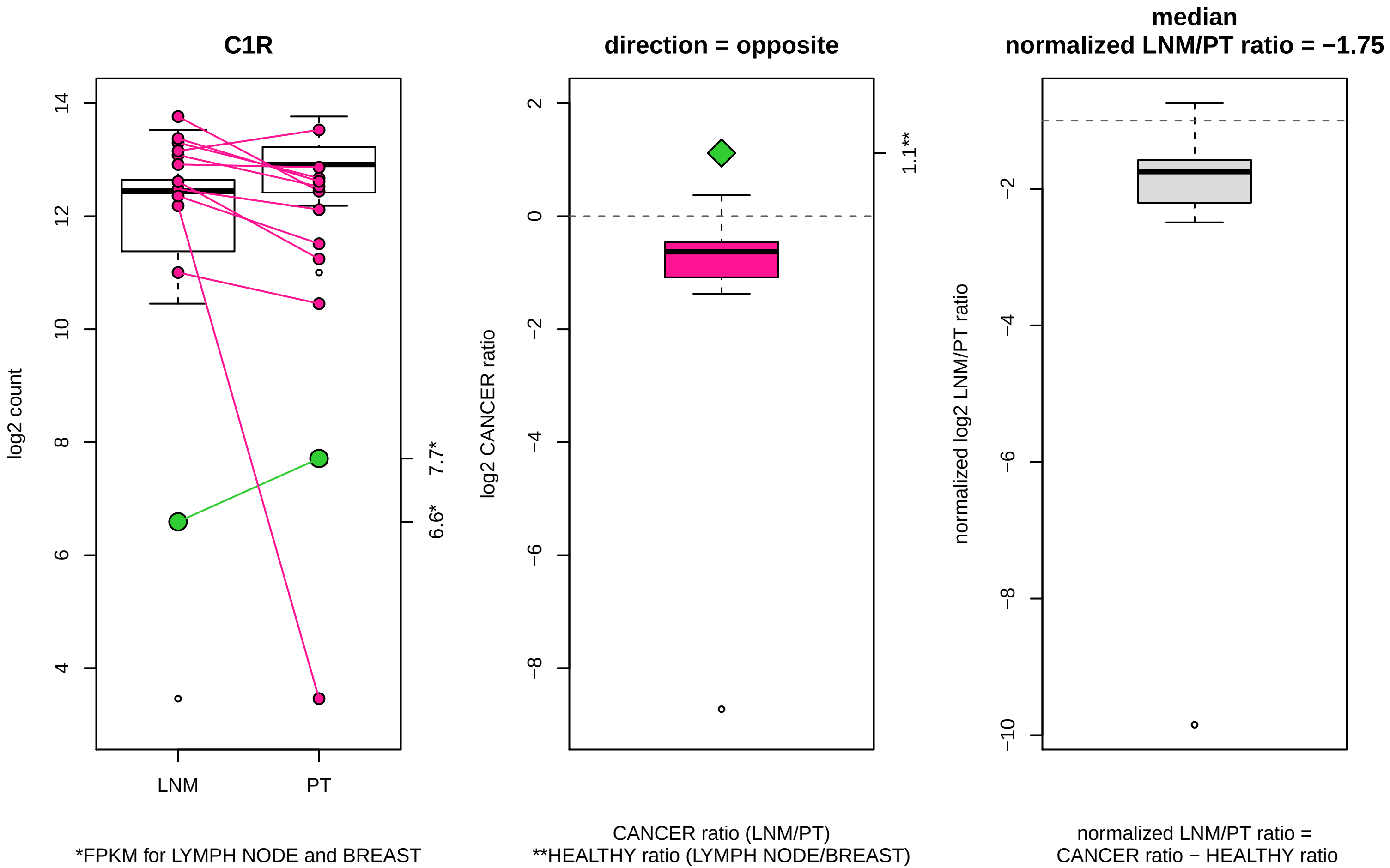

C1R
log2 count
14
12
10
8
6
4
7.7*
6.6*
LNM
PT
*FPKM for LYMPH NODE and BREAST
direction = opposite
log2 CANCER ratio
2
0
−2
−4
−6
−8
1.1**
CANCER ratio (LNM/PT)
**HEALTHY ratio (LYMPH NODE/BREAST)
median
normalized LNM/PT ratio = −1.75
normalized log2 LNM/PT ratio
−2
−4
−6
−8
−10
normalized LNM/PT ratio =
CANCER ratio − HEALTHY ratio

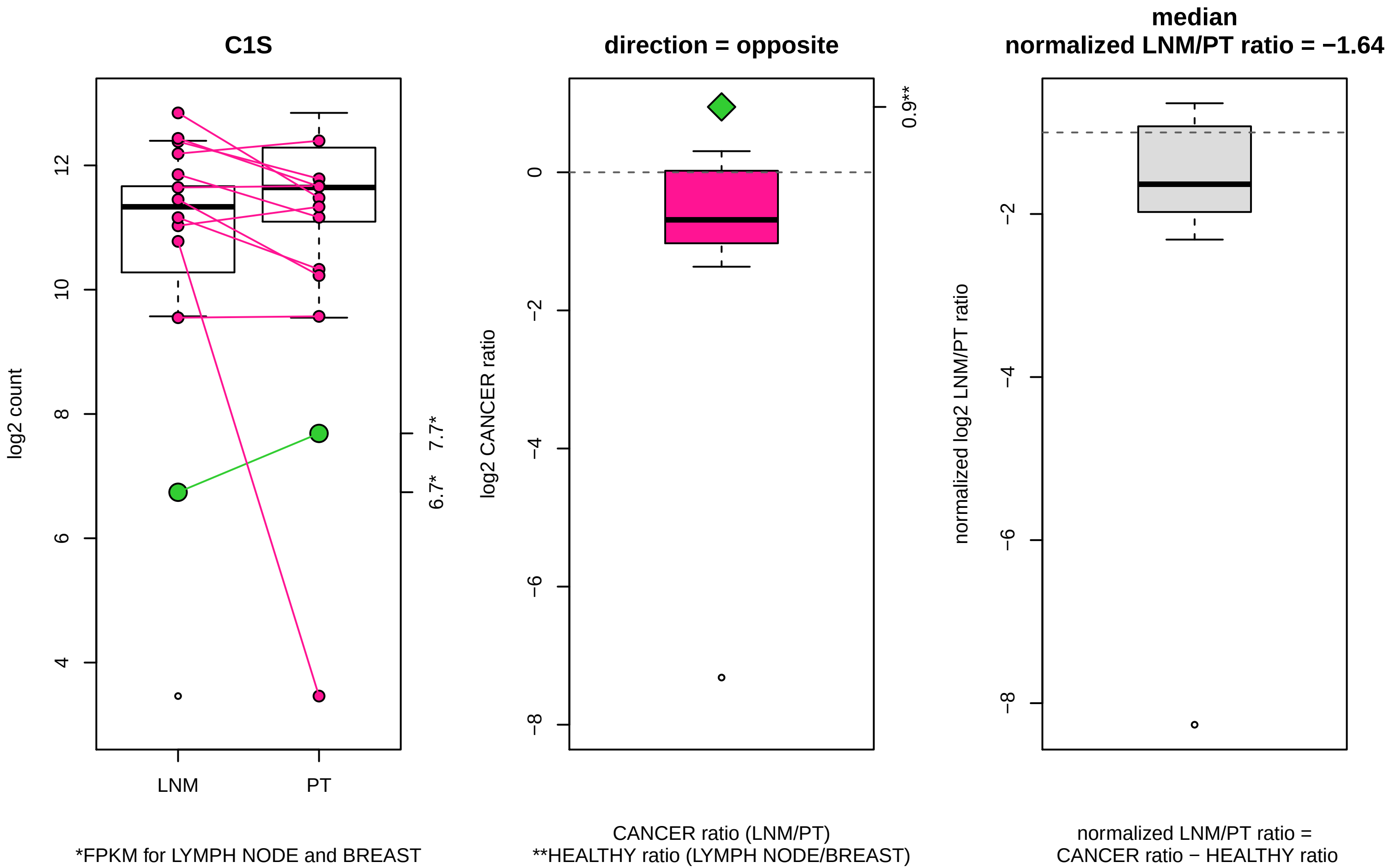

C1S
log2 count
LNM
PT
6.7*
7.7*
*FPKM for LYMPH NODE and BREAST
direction = opposite
log2 CANCER ratio
0.9**
CANCER ratio (LNM/PT)
**HEALTHY ratio (LYMPH NODE/BREAST)
median
normalized LNM/PT ratio = −1.64
normalized log2 LNM/PT ratio
normalized LNM/PT ratio =
CANCER ratio − HEALTHY ratio

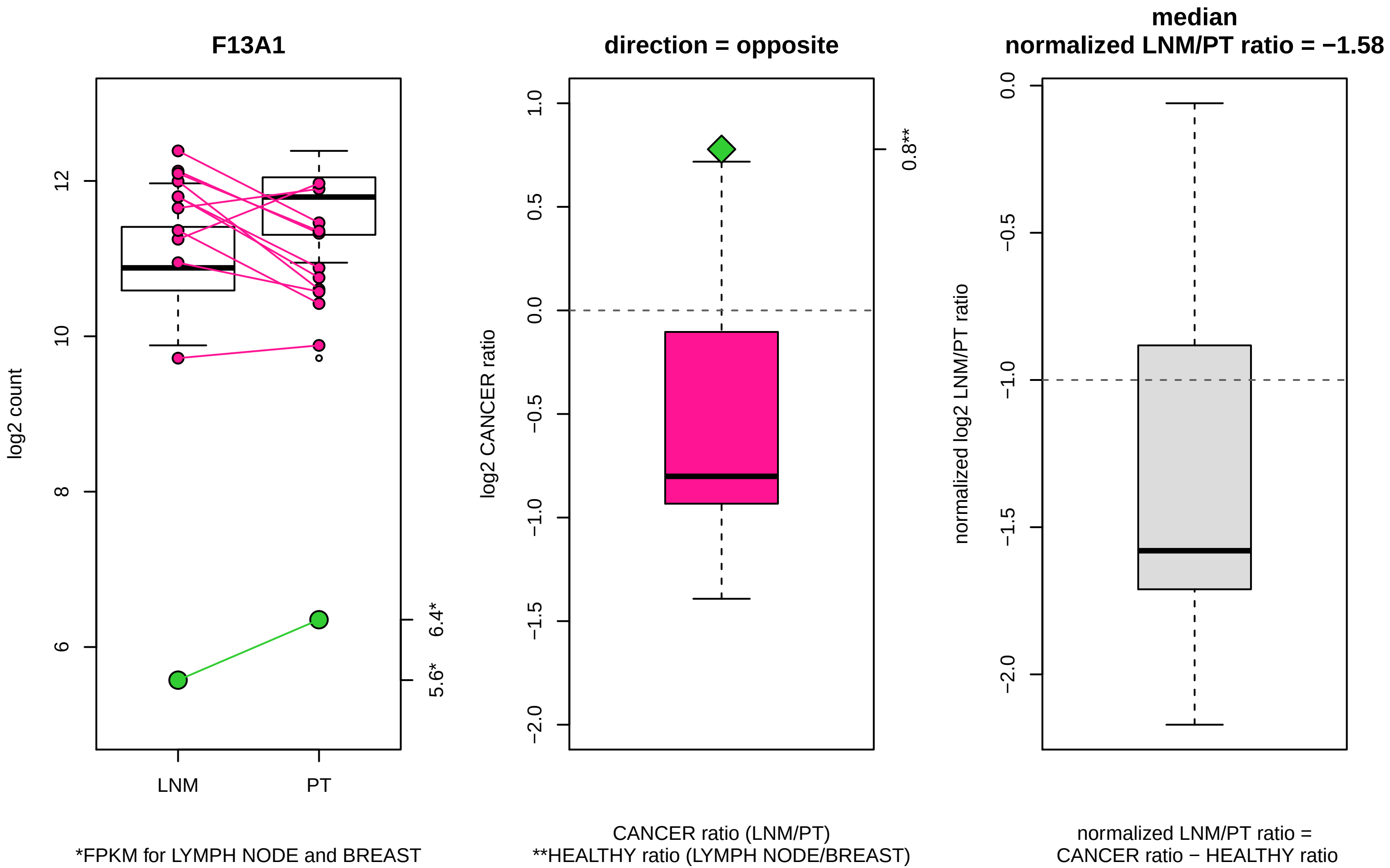
F13A1
log2 count
12
10
8
6
6.4*
5.6*
LNM
PT
*FPKM for LYMPH NODE and BREAST
direction = opposite
log2 CANCER ratio
1.0
0.5
0.0
−0.5
−1.0
−1.5
−2.0
0.8**
CANCER ratio (LNM/PT)
**HEALTHY ratio (LYMPH NODE/BREAST)
median
normalized LNM/PT ratio = −1.58
normalized log2 LNM/PT ratio
0.0
−0.5
−1.0
−1.5
−2.0
normalized LNM/PT ratio =
CANCER ratio − HEALTHY ratio

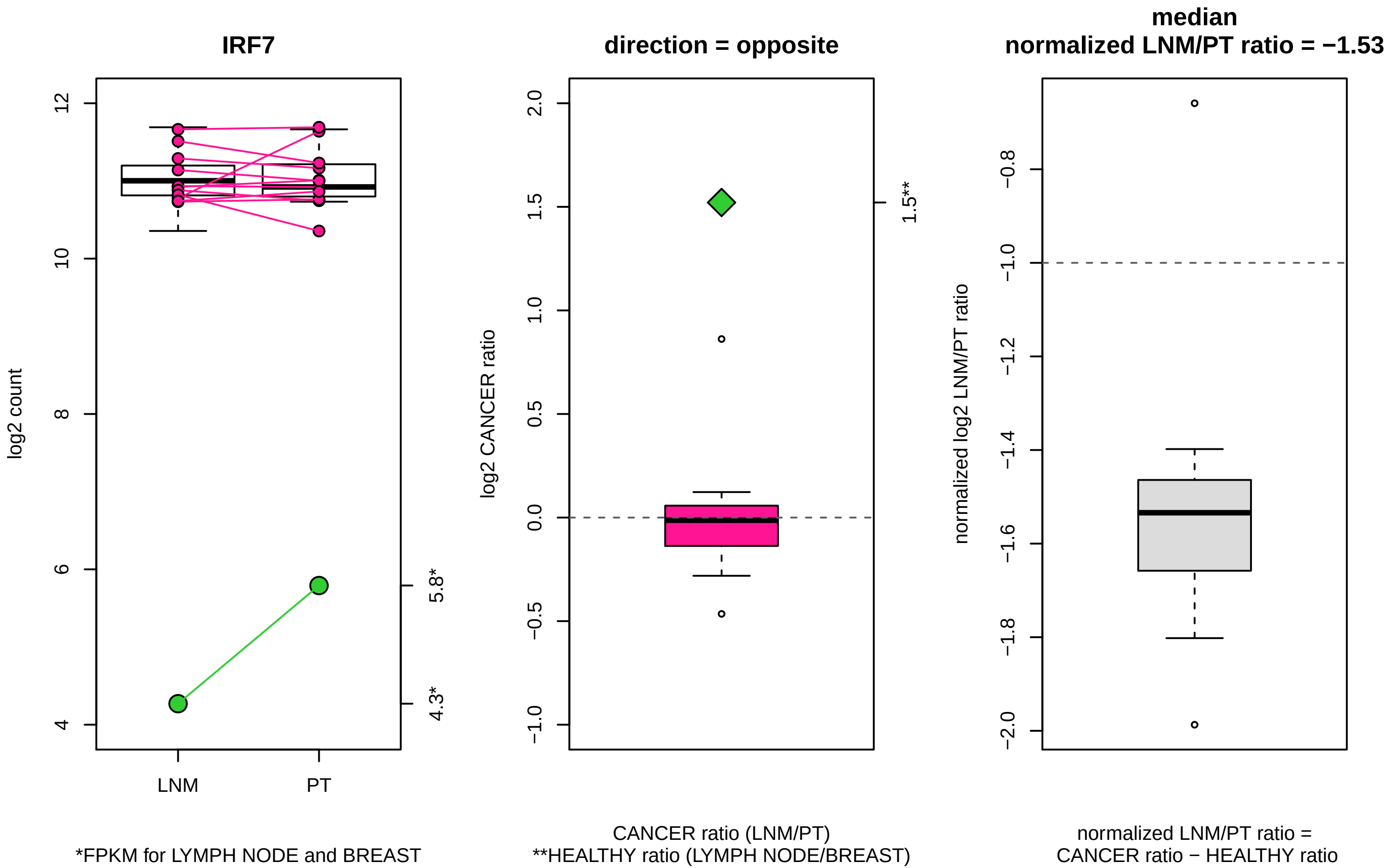
IRF7
direction = opposite
median
normalized LNM/PT ratio = −1.53
log2 count
12
10
8
6
4
5.8*
4.3*
LNM
PT
*FPKM for LYMPH NODE and BREAST
log2 CANCER ratio
2.0
1.5
1.0
0.5
0.0
−0.5
−1.0
1.5**
CANCER ratio (LNM/PT)
**HEALTHY ratio (LYMPH NODE/BREAST)
normalized log2 LNM/PT ratio
−0.8
−1.0
−1.2
−1.4
−1.6
−1.8
−2.0
normalized LNM/PT ratio =
CANCER ratio − HEALTHY ratio

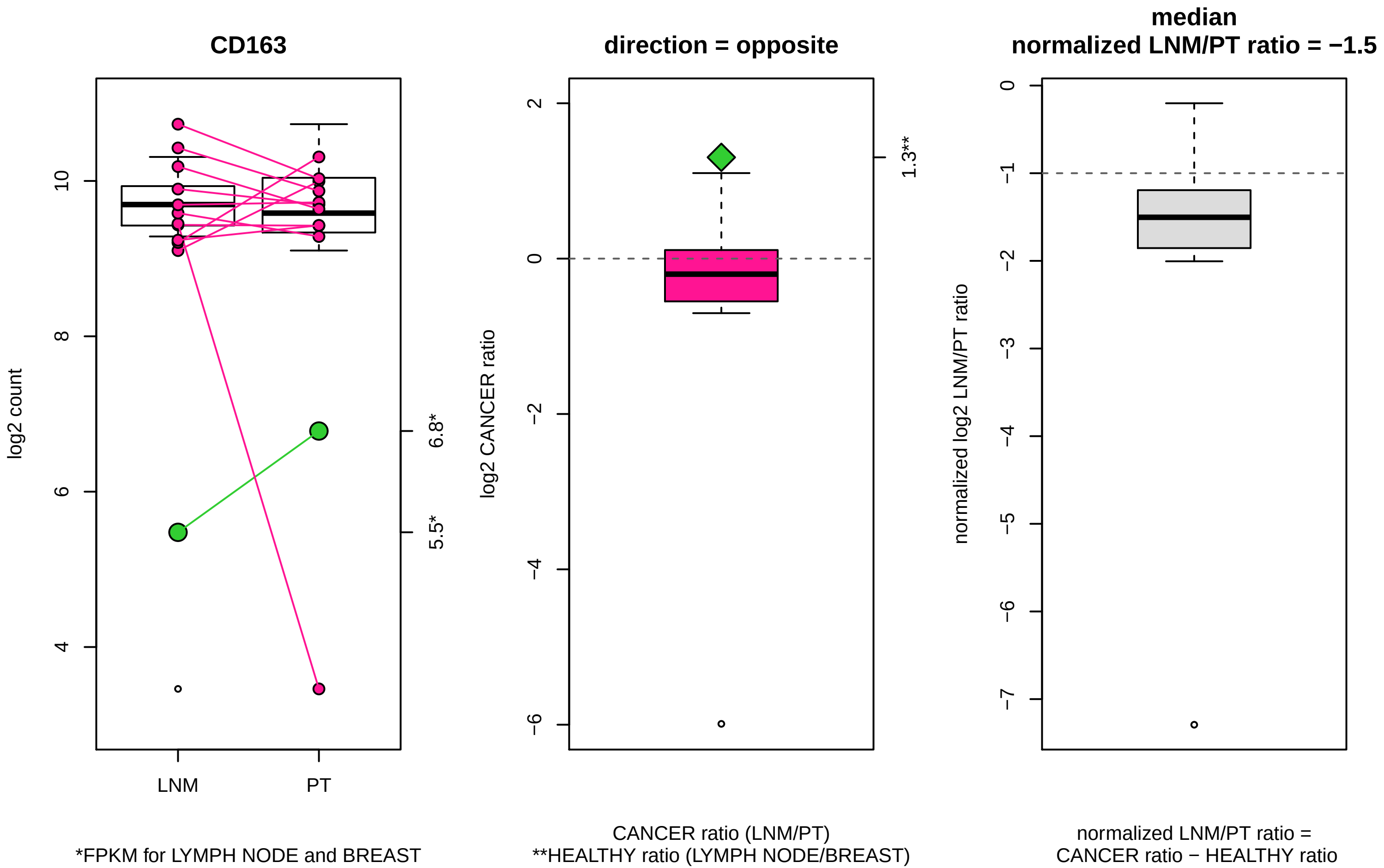
CD163
log2 count
10
8
6
4
LNM
PT
6.8*
5.5*
*FPKM for LYMPH NODE and BREAST
direction = opposite
log2 CANCER ratio
2
0
−2
−4
−6
1.3**
CANCER ratio (LNM/PT)
**HEALTHY ratio (LYMPH NODE/BREAST)
median
normalized LNM/PT ratio = −1.5
normalized log2 LNM/PT ratio
0
−1
−2
−3
−4
−5
−6
−7
normalized LNM/PT ratio =
CANCER ratio − HEALTHY ratio

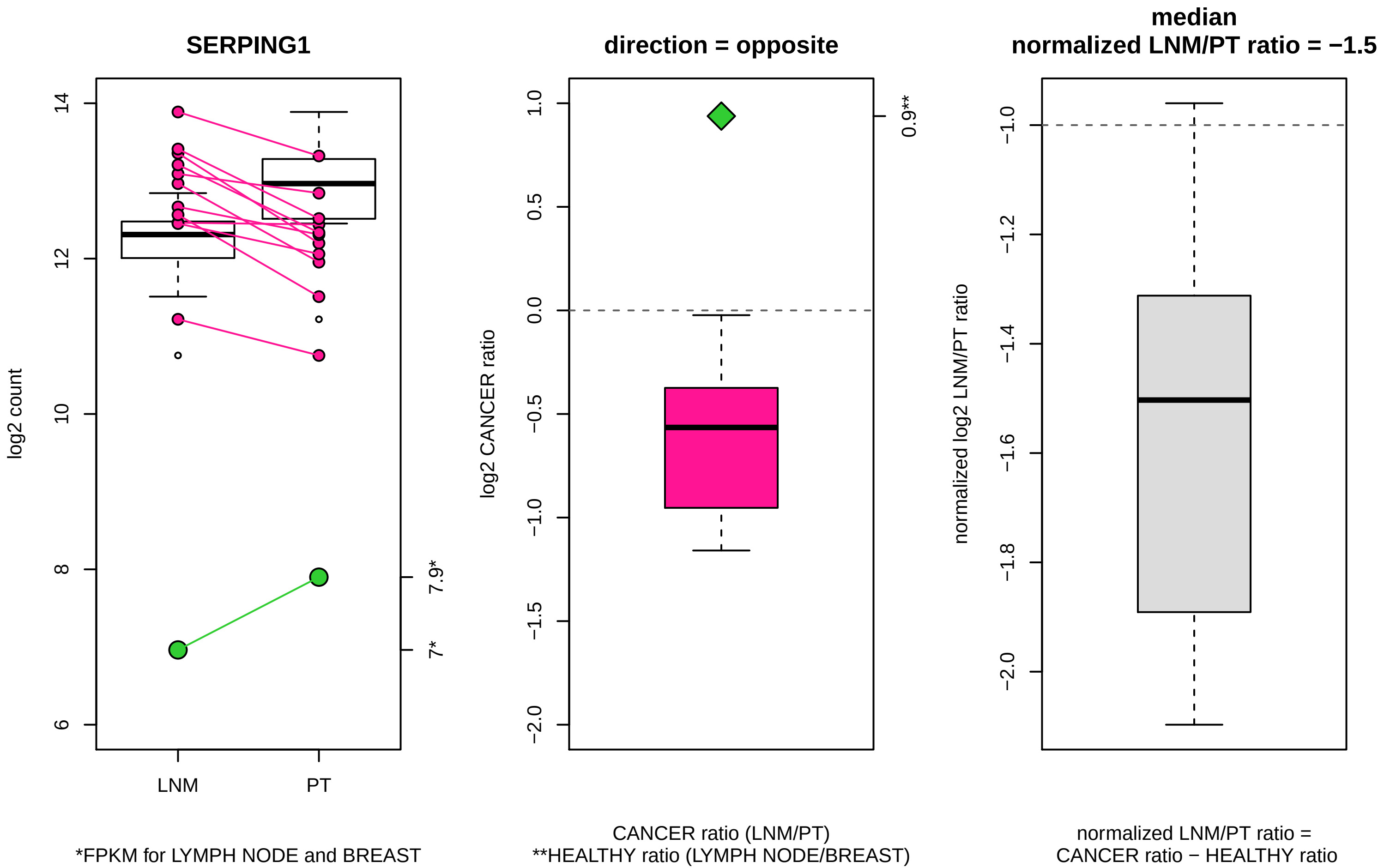
SERPING1
log2 count
14
12
10
8
6
LNM
PT
7.9*
7*
*FPKM for LYMPH NODE and BREAST
direction = opposite
log2 CANCER ratio
1.0
0.5
0.0
−0.5
−1.0
−1.5
−2.0
0.9**
CANCER ratio (LNM/PT)
**HEALTHY ratio (LYMPH NODE/BREAST)
median
normalized LNM/PT ratio = −1.5
normalized log2 LNM/PT ratio
−1.0
−1.2
−1.4
−1.6
−1.8
−2.0
normalized LNM/PT ratio =
CANCER ratio − HEALTHY ratio

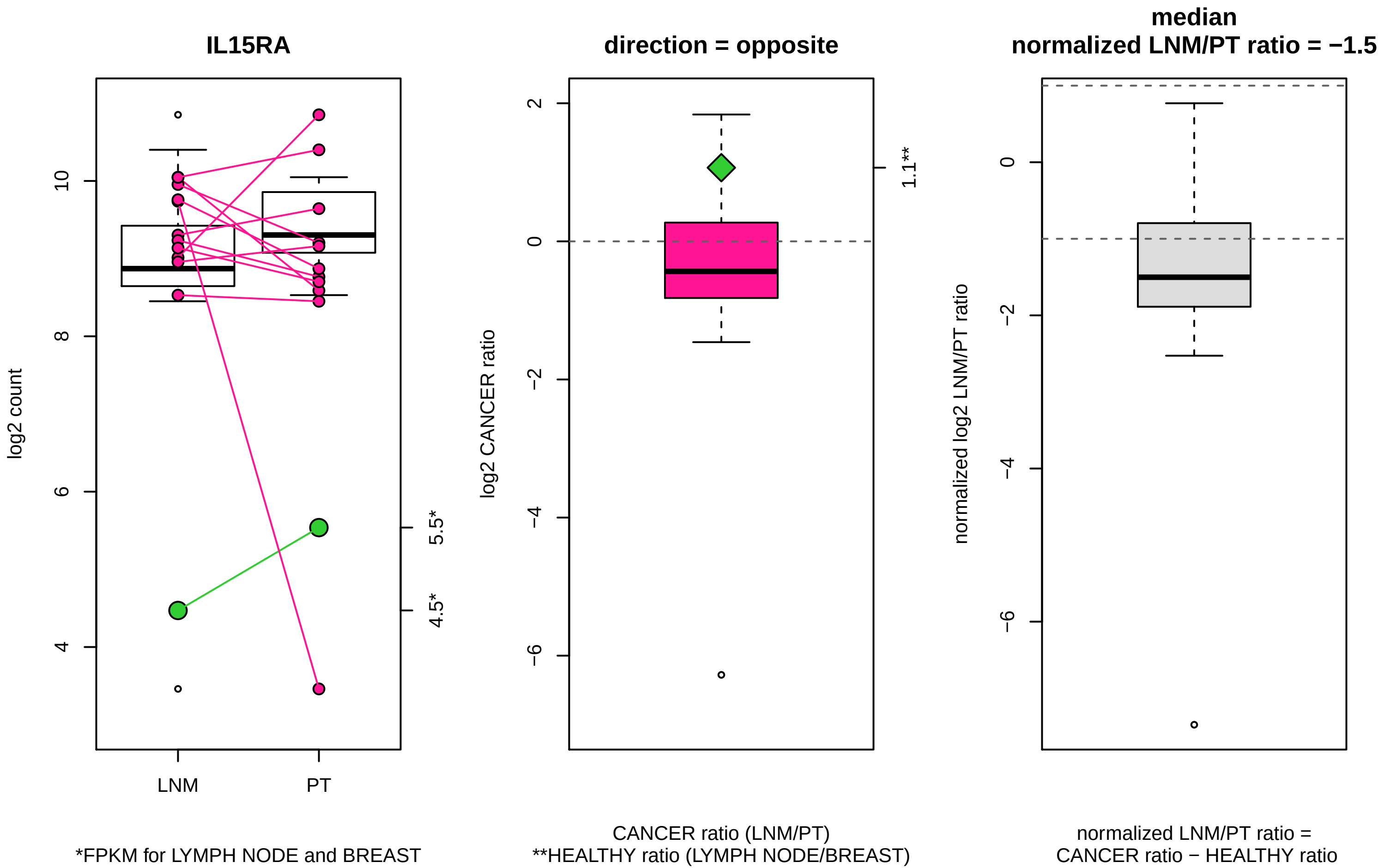
IL15RA
direction = opposite
median
normalized LNM/PT ratio = −1.5
log2 count
10
8
6
4
LNM
PT
5.5*
4.5*
log2 CANCER ratio
2
0
−2
−4
−6
1.1**
normalized log2 LNM/PT ratio
0
−2
−4
−6
*FPKM for LYMPH NODE and BREAST
CANCER ratio (LNM/PT)
**HEALTHY ratio (LYMPH NODE/BREAST)
normalized LNM/PT ratio =
CANCER ratio − HEALTHY ratio

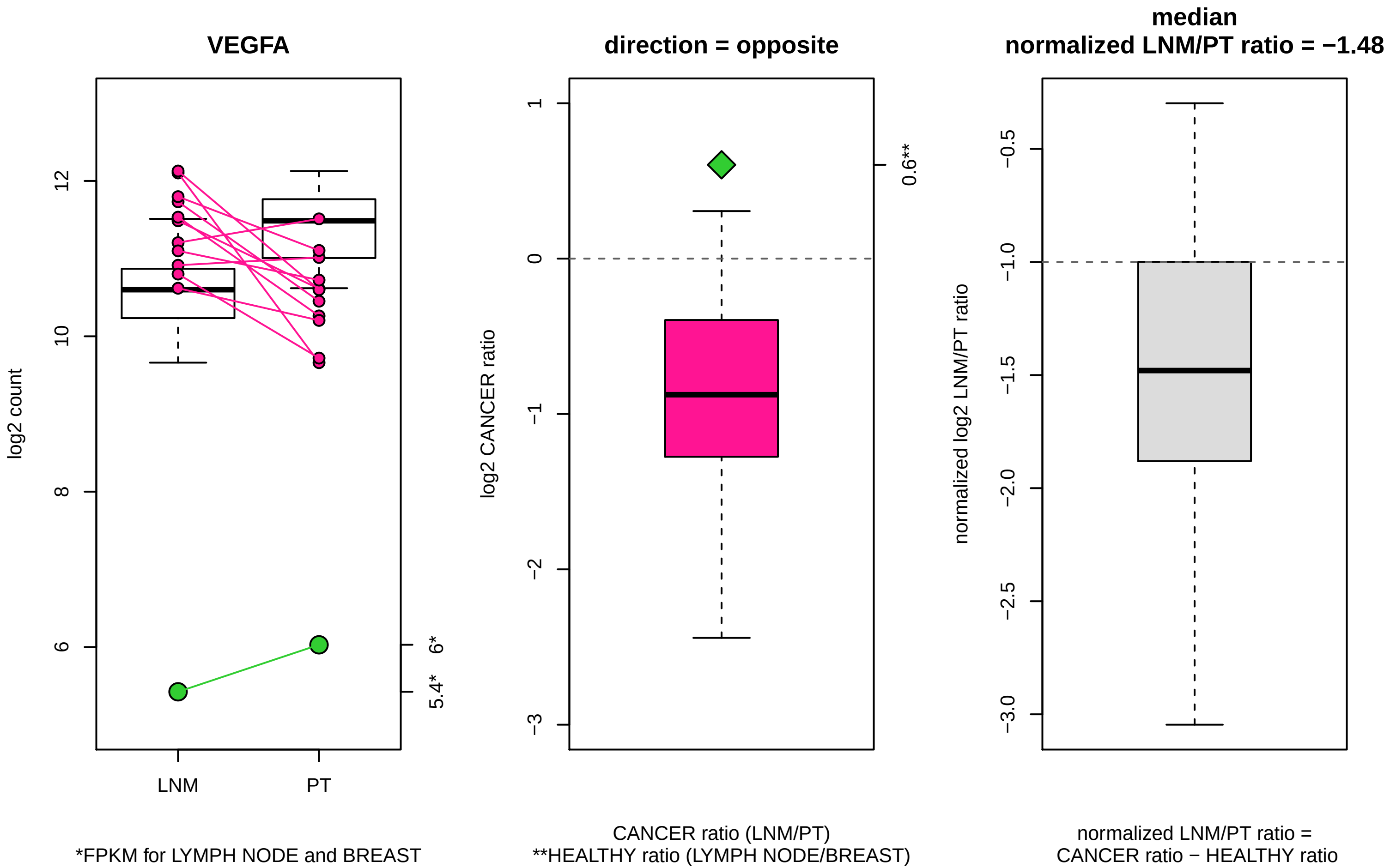
VEGFA
log2 count
12
10
8
6
LNM
PT
6*
5.4*
*FPKM for LYMPH NODE and BREAST
direction = opposite
log2 CANCER ratio
1
0
−1
−2
−3
0.6**
CANCER ratio (LNM/PT)
**HEALTHY ratio (LYMPH NODE/BREAST)
median
normalized LNM/PT ratio = −1.48
normalized log2 LNM/PT ratio
−0.5
−1.0
−1.5
−2.0
−2.5
−3.0
normalized LNM/PT ratio =
CANCER ratio − HEALTHY ratio

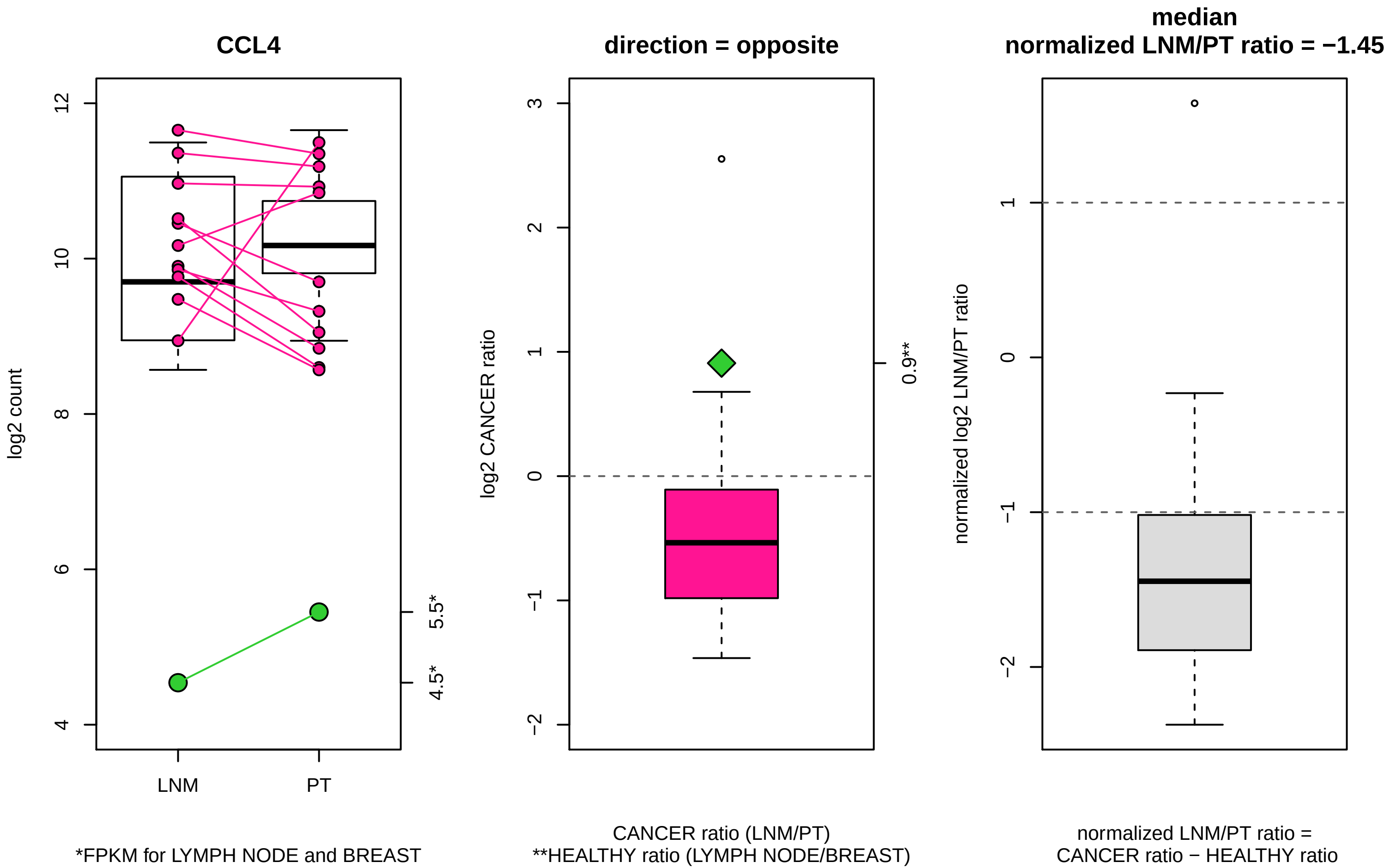

CCL4
direction = opposite
median
normalized LNM/PT ratio = −1.45
log2 count
12
10
8
6
4
LNM
PT
5.5*
4.5*
*FPKM for LYMPH NODE and BREAST
log2 CANCER ratio
3
2
1
0
−1
−2
0.9**
CANCER ratio (LNM/PT)
**HEALTHY ratio (LYMPH NODE/BREAST)
normalized log2 LNM/PT ratio
1
0
−1
−2
normalized LNM/PT ratio =
CANCER ratio − HEALTHY ratio

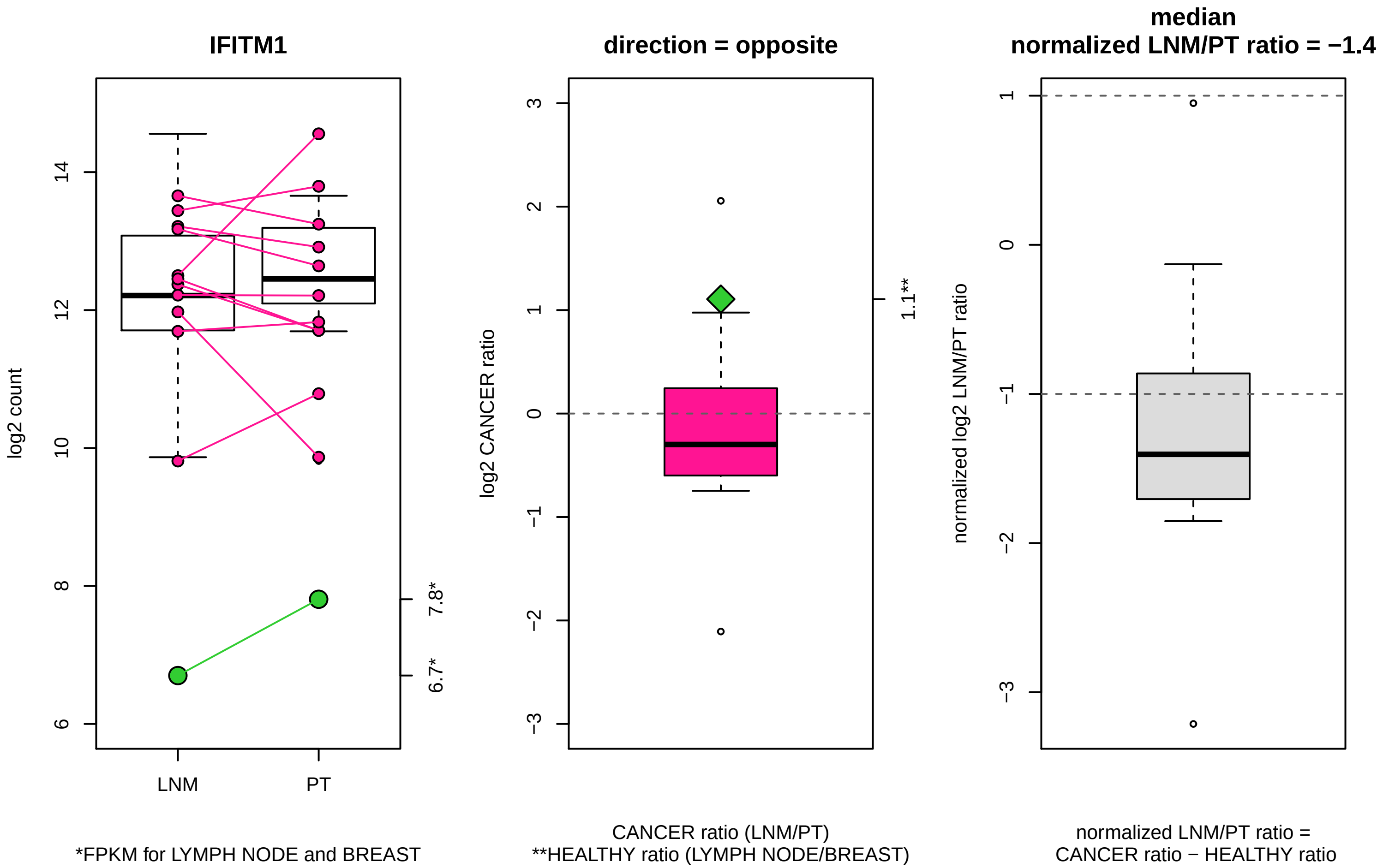

*FPKM for LYMPH NODE and BREAST

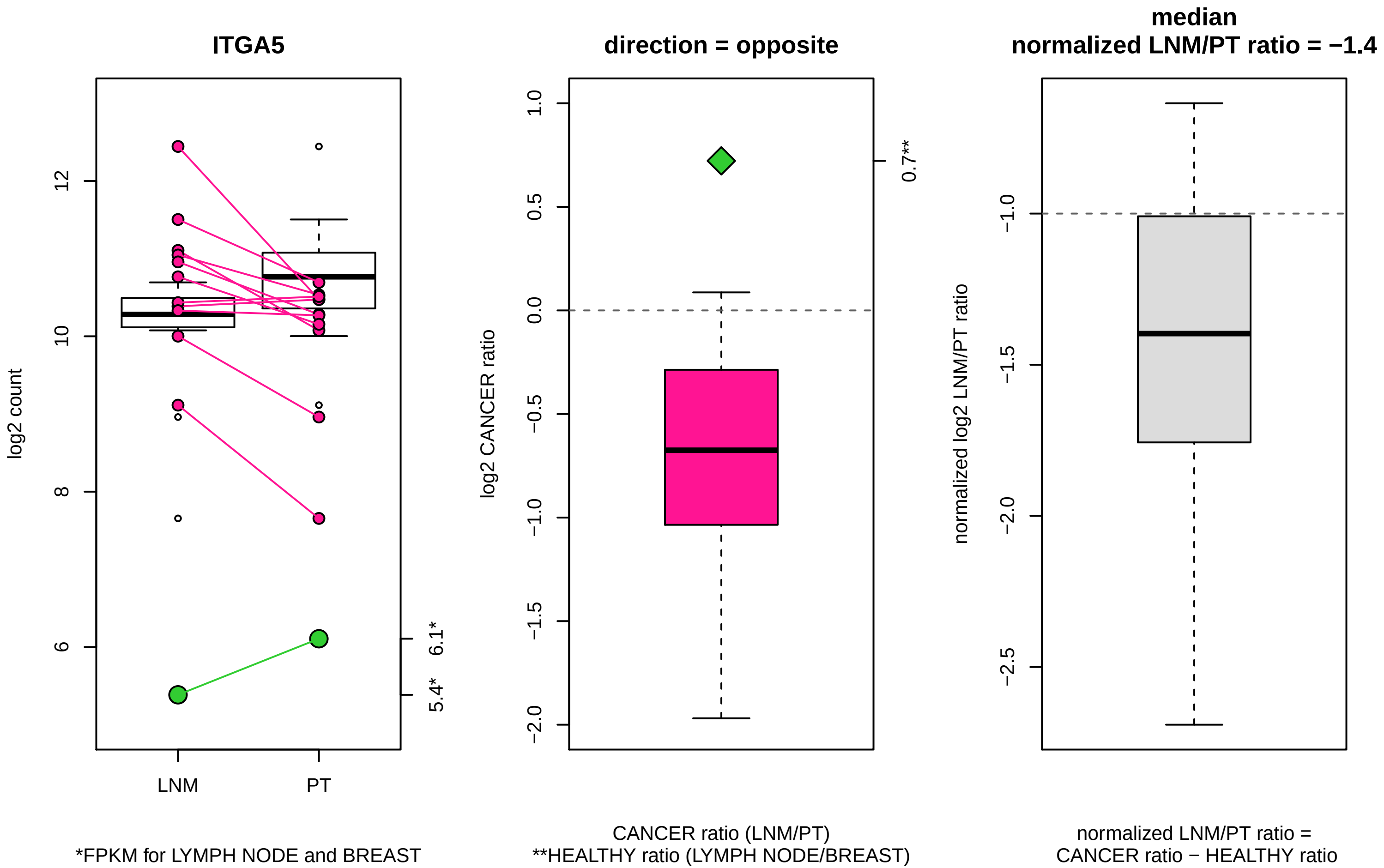
ITGA5
log2 count
12
10
8
6
LNM
PT
6.1*
5.4*
*FPKM for LYMPH NODE and BREAST
direction = opposite
log2 CANCER ratio
1.0
0.5
0.0
−0.5
−1.0
−1.5
−2.0
0.7**
CANCER ratio (LNM/PT)
**HEALTHY ratio (LYMPH NODE/BREAST)
median
normalized LNM/PT ratio = −1.4
normalized log2 LNM/PT ratio
−1.0
−1.5
−2.0
−2.5
normalized LNM/PT ratio =
CANCER ratio − HEALTHY ratio

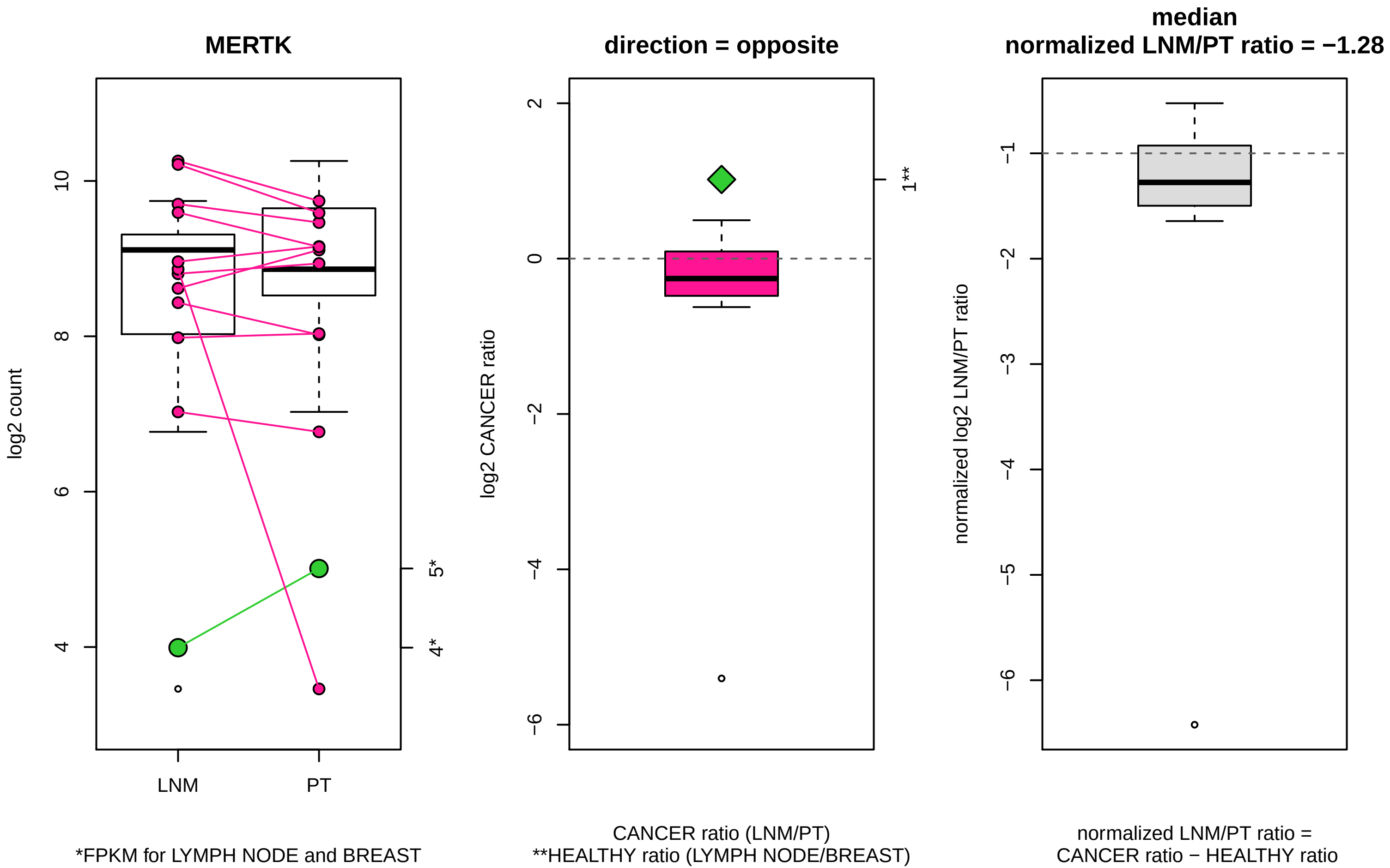

MERTK
log2 count
10
8
6
4
5*
4*
LNM
PT
*FPKM for LYMPH NODE and BREAST
direction = opposite
log2 CANCER ratio
2
0
−2
−4
−6
1**
CANCER ratio (LNM/PT)
**HEALTHY ratio (LYMPH NODE/BREAST)
median
normalized LNM/PT ratio = −1.28
normalized log2 LNM/PT ratio
−1
−2
−3
−4
−5
−6
normalized LNM/PT ratio =
CANCER ratio − HEALTHY ratio

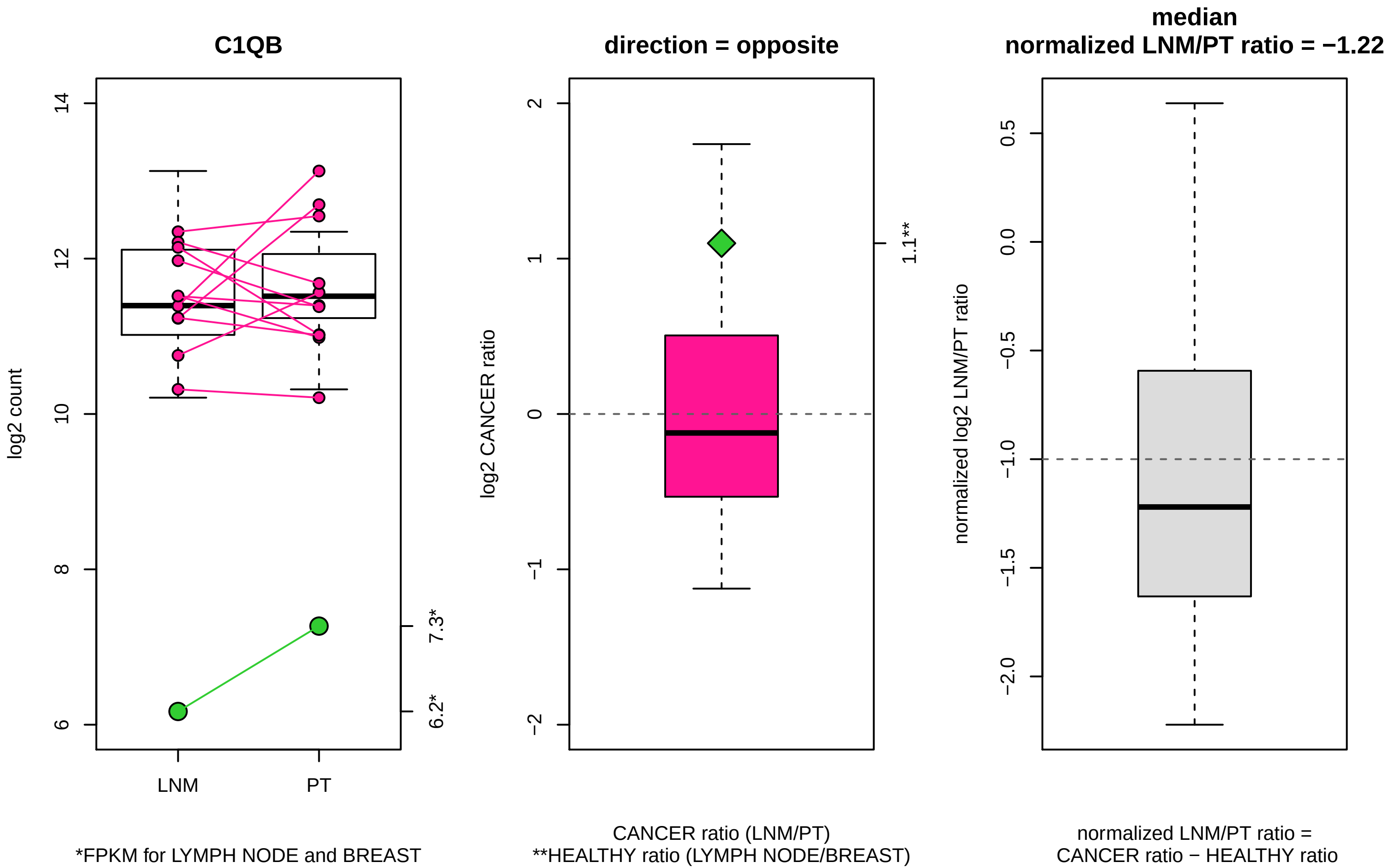

C1QB
log2 count
14
12
10
8
6
7.3*
6.2*
LNM
PT
*FPKM for LYMPH NODE and BREAST
direction = opposite
log2 CANCER ratio
2
1
0
−1
−2
1.1**
CANCER ratio (LNM/PT)
**HEALTHY ratio (LYMPH NODE/BREAST)
median
normalized LNM/PT ratio = −1.22
normalized log2 LNM/PT ratio
0.5
0.0
−0.5
−1.0
−1.5
−2.0
normalized LNM/PT ratio =
CANCER ratio − HEALTHY ratio

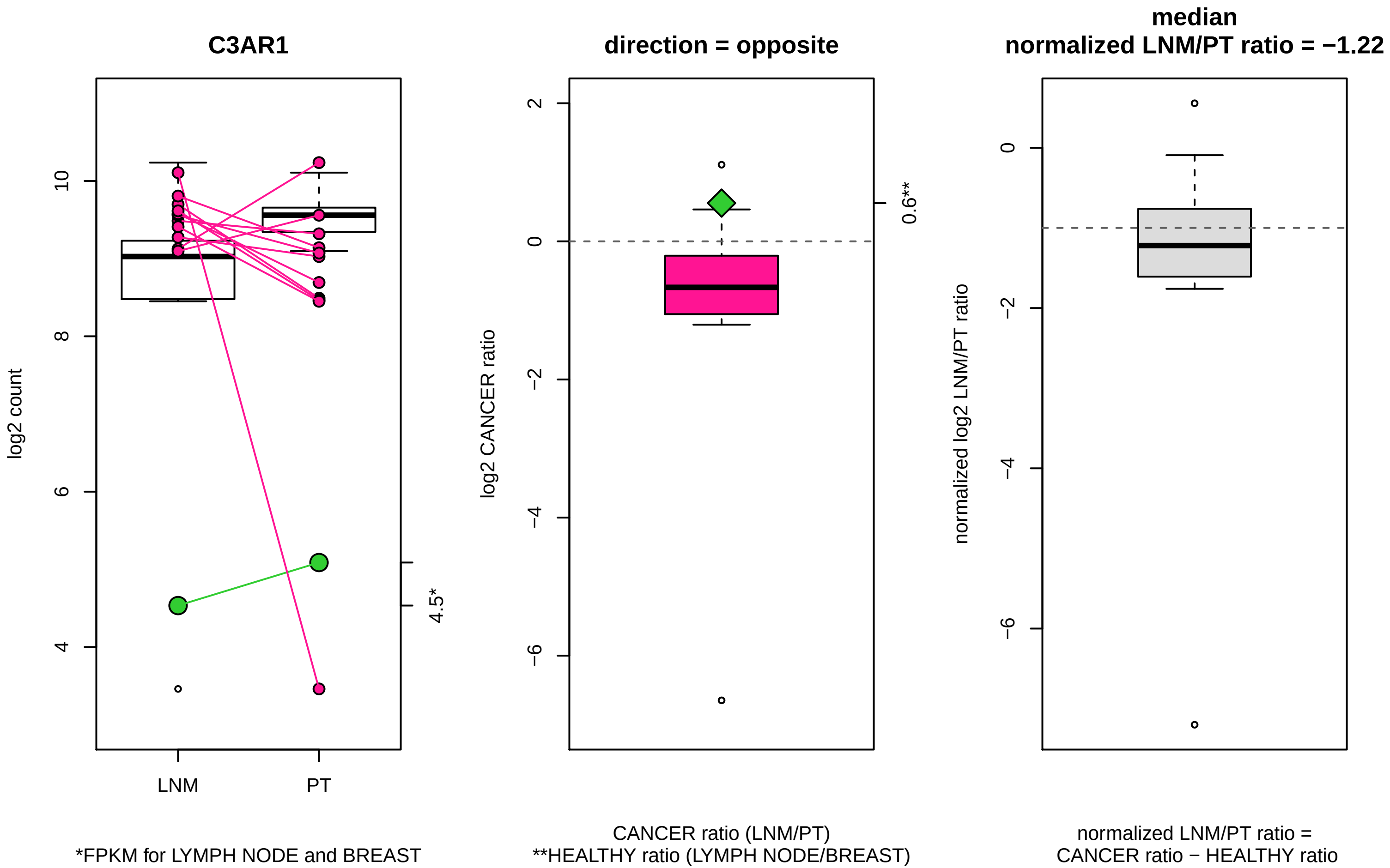
C3AR1
log2 count
10
8
6
4
LNM
PT
4.5*
*FPKM for LYMPH NODE and BREAST
direction = opposite
log2 CANCER ratio
2
0
−2
−4
−6
0.6**
CANCER ratio (LNM/PT)
**HEALTHY ratio (LYMPH NODE/BREAST)
median
normalized LNM/PT ratio = −1.22
normalized log2 LNM/PT ratio
0
−2
−4
−6
normalized LNM/PT ratio =
CANCER ratio − HEALTHY ratio

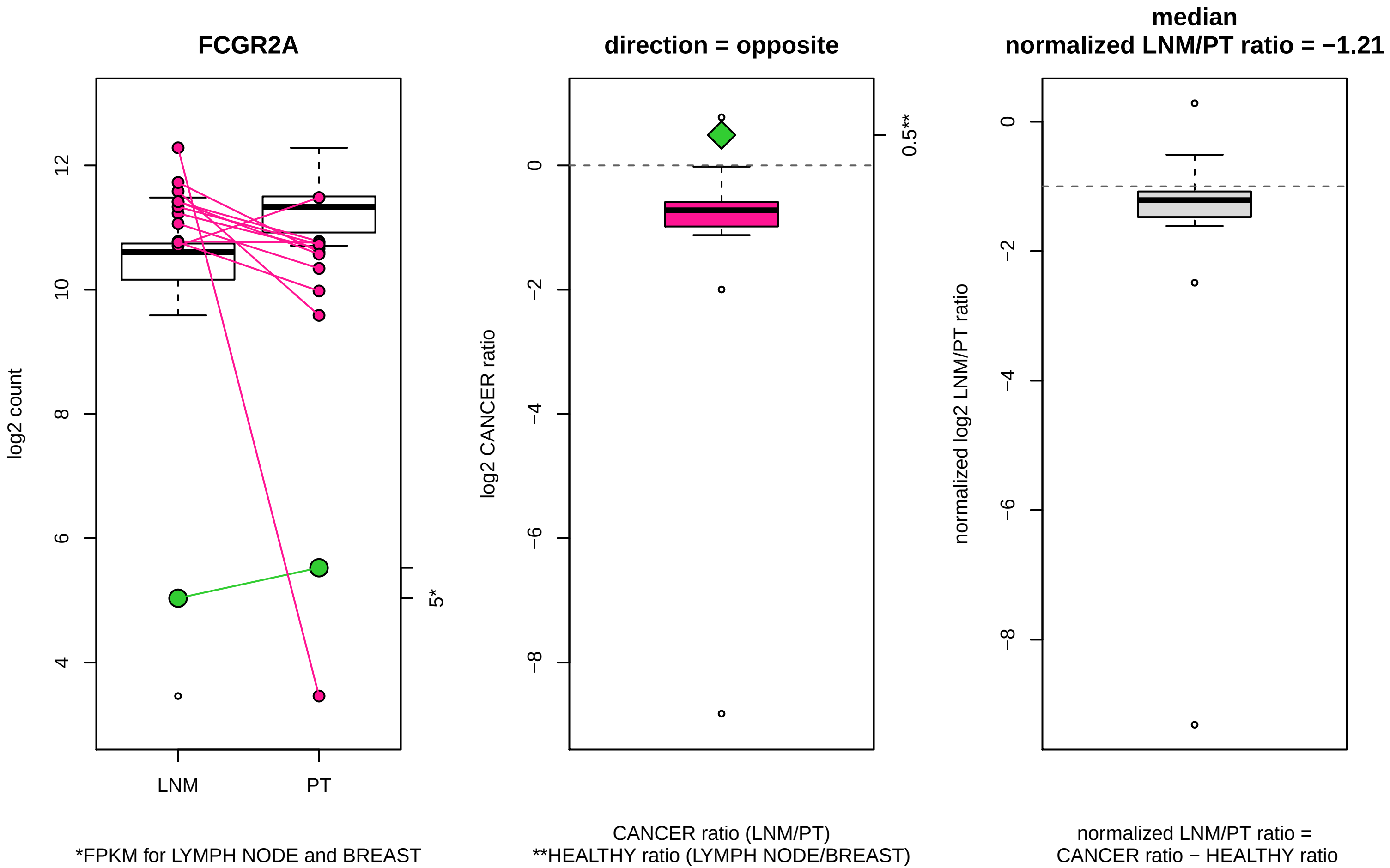
FCGR2A
log2 count
LNM
PT
5*
*FPKM for LYMPH NODE and BREAST
direction = opposite
log2 CANCER ratio
0.5**
CANCER ratio (LNM/PT)
**HEALTHY ratio (LYMPH NODE/BREAST)
median
normalized LNM/PT ratio = −1.21
normalized log2 LNM/PT ratio
normalized LNM/PT ratio =
CANCER ratio − HEALTHY ratio

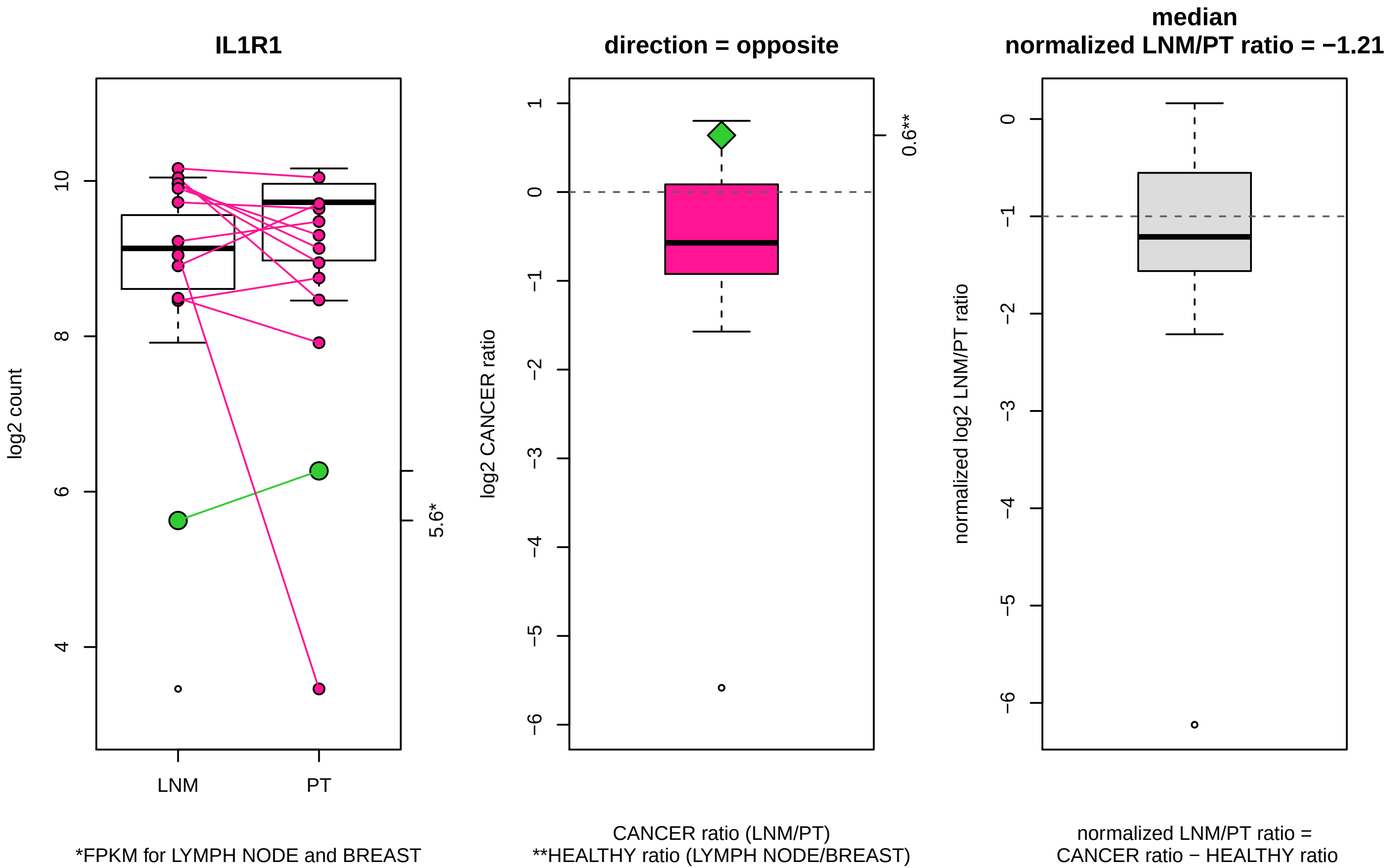
IL1R1
log2 count
10
8
6
4
LNM
PT
5.6*
*FPKM for LYMPH NODE and BREAST
direction = opposite
log2 CANCER ratio
1
0
−1
−2
−3
−4
−5
−6
0.6**
CANCER ratio (LNM/PT)
**HEALTHY ratio (LYMPH NODE/BREAST)
median
normalized LNM/PT ratio = −1.21
normalized log2 LNM/PT ratio
0
−1
−2
−3
−4
−5
−6
normalized LNM/PT ratio =
CANCER ratio − HEALTHY ratio

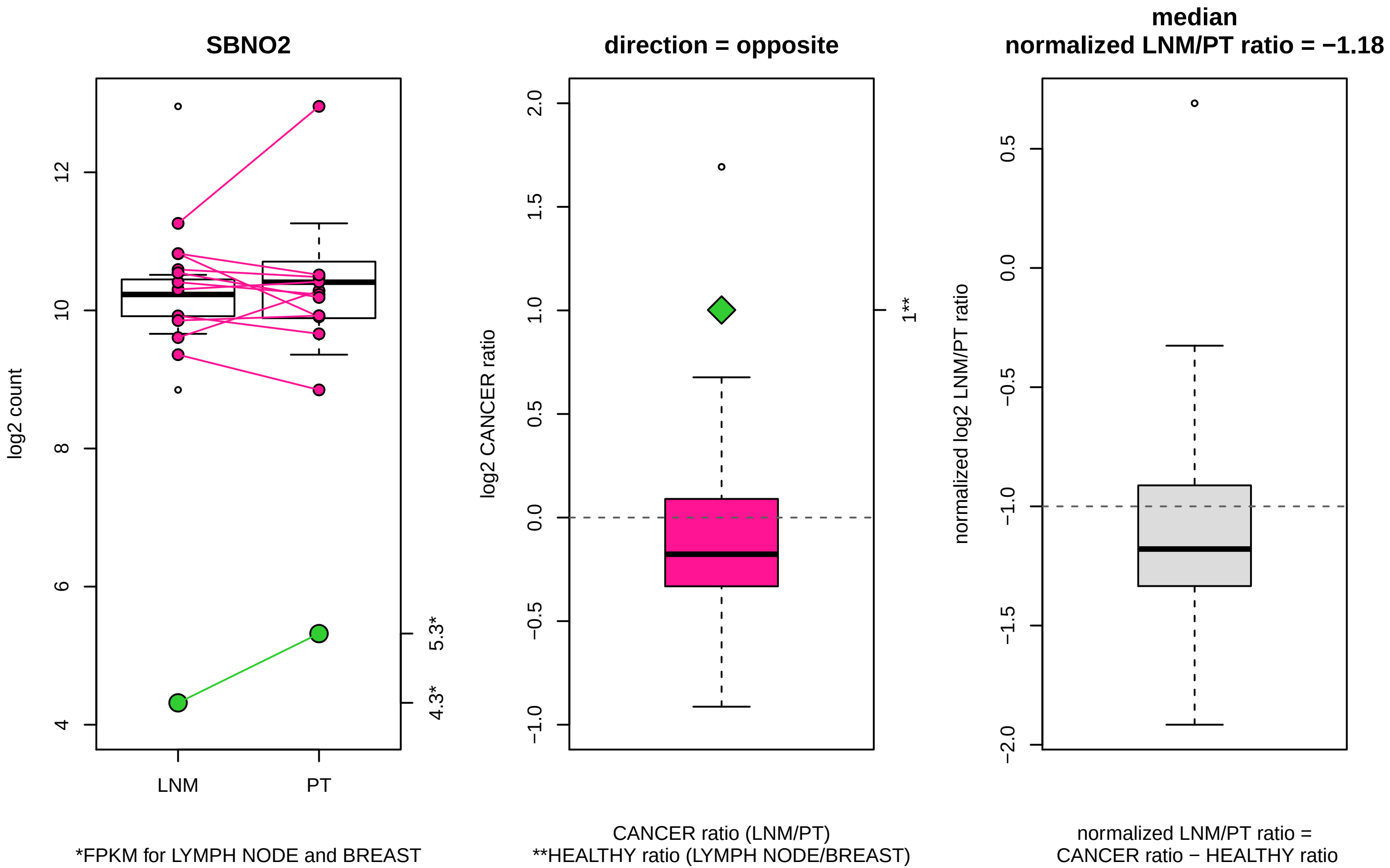
SBNO2
direction = opposite
median
normalized LNM/PT ratio = −1.18
log2 count
12
10
8
6
4
LNM
PT
5.3*
4.3*
log2 CANCER ratio
2.0
1.5
1.0
0.5
0.0
−0.5
−1.0
1**
normalized log2 LNM/PT ratio
0.5
0.0
−0.5
−1.0
−1.5
−2.0
*FPKM for LYMPH NODE and BREAST
CANCER ratio (LNM/PT)
**HEALTHY ratio (LYMPH NODE/BREAST)
normalized LNM/PT ratio =
CANCER ratio − HEALTHY ratio

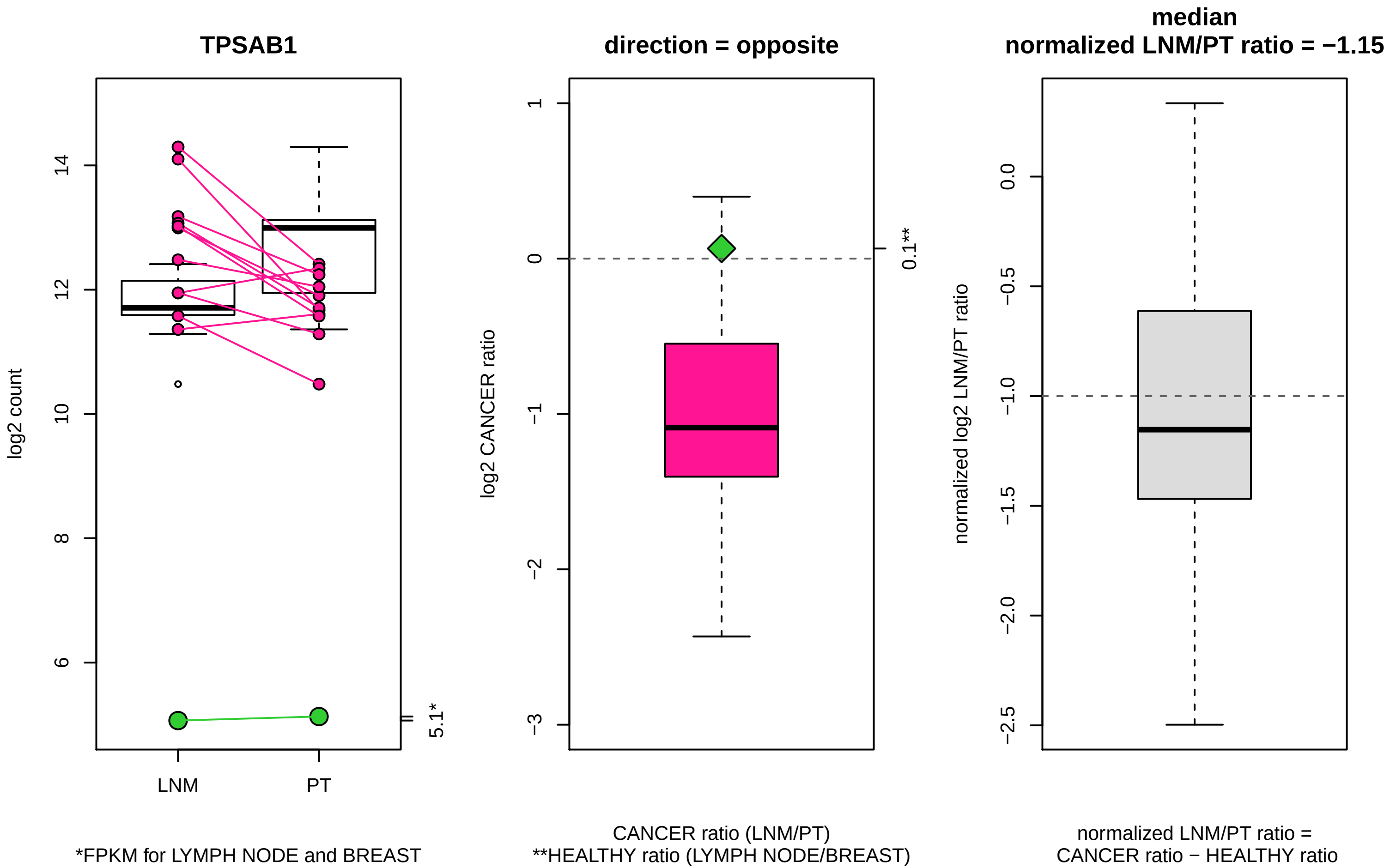
TPSAB1
log2 count
14
12
10
8
6
LNM
PT
5.1*
*FPKM for LYMPH NODE and BREAST
direction = opposite
log2 CANCER ratio
1
0
−1
−2
−3
0.1**
CANCER ratio (LNM/PT)
**HEALTHY ratio (LYMPH NODE/BREAST)
median
normalized LNM/PT ratio = −1.15
normalized log2 LNM/PT ratio
0.0
−0.5
−1.0
−1.5
−2.0
−2.5
normalized LNM/PT ratio =
CANCER ratio − HEALTHY ratio

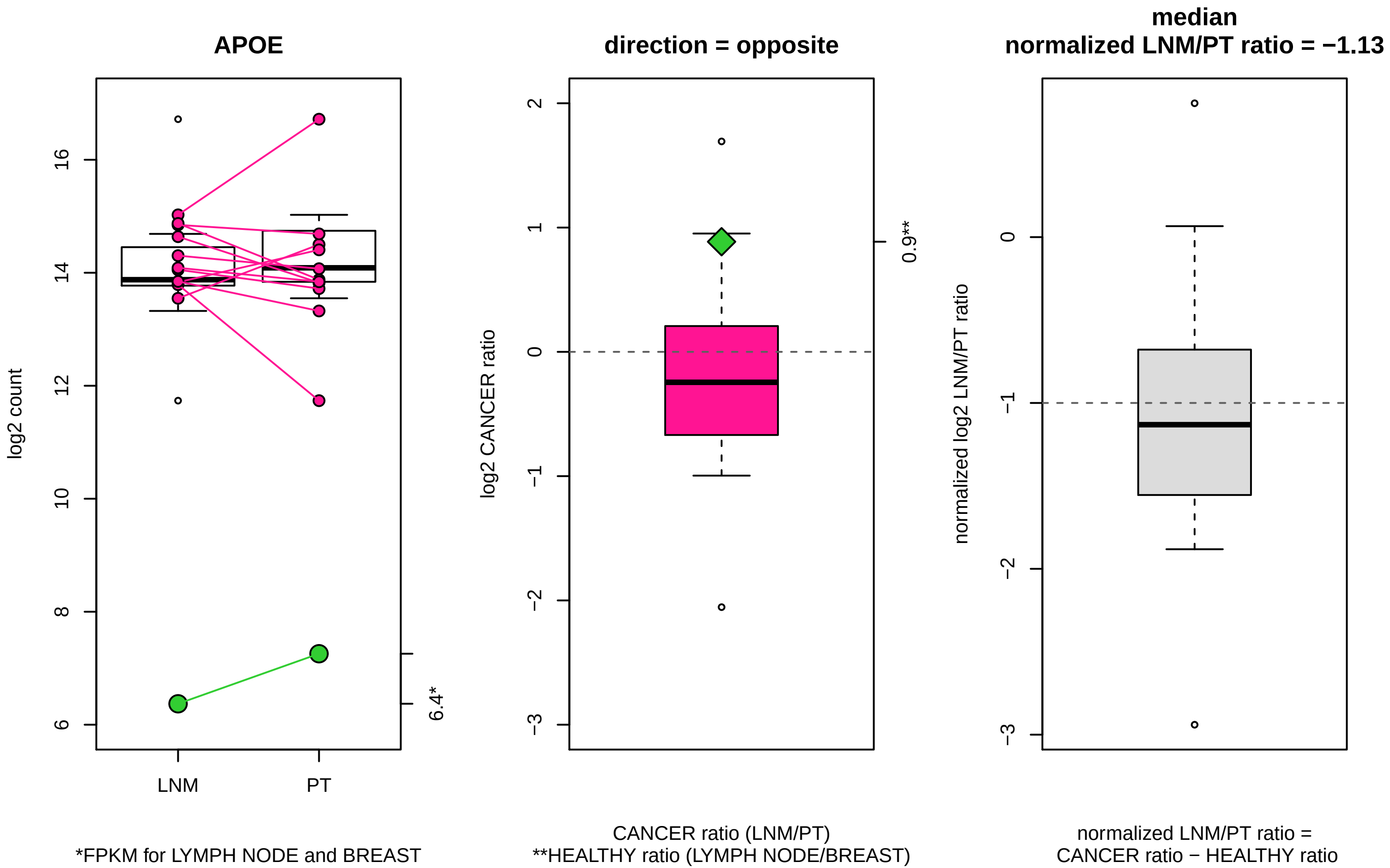

APOE
log2 count
16
14
12
10
8
6
LNM
PT
6.4*
*FPKM for LYMPH NODE and BREAST
direction = opposite
log2 CANCER ratio
2
1
0
−1
−2
−3
0.9**
CANCER ratio (LNM/PT)
**HEALTHY ratio (LYMPH NODE/BREAST)
median
normalized LNM/PT ratio = −1.13
normalized log2 LNM/PT ratio
0
−1
−2
−3
normalized LNM/PT ratio =
CANCER ratio − HEALTHY ratio

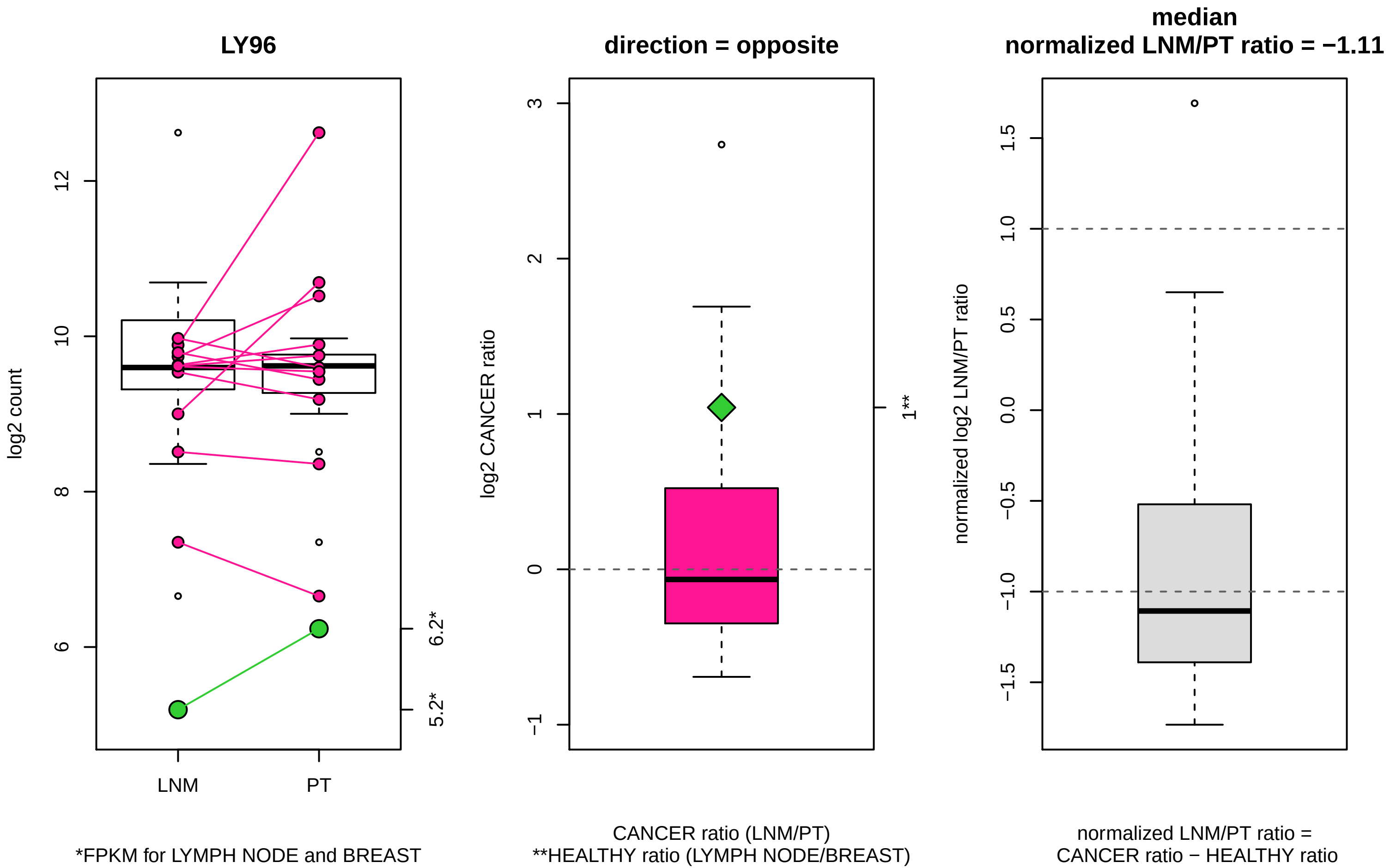

LY96
log2 count
12
10
8
6
LNM
PT
6.2*
5.2*
*FPKM for LYMPH NODE and BREAST
direction = opposite
log2 CANCER ratio
3
2
1
0
−1
1**
CANCER ratio (LNM/PT)
**HEALTHY ratio (LYMPH NODE/BREAST)
median
normalized LNM/PT ratio = −1.11
normalized log2 LNM/PT ratio
1.5
1.0
0.5
0.0
−0.5
−1.0
−1.5
normalized LNM/PT ratio =
CANCER ratio − HEALTHY ratio

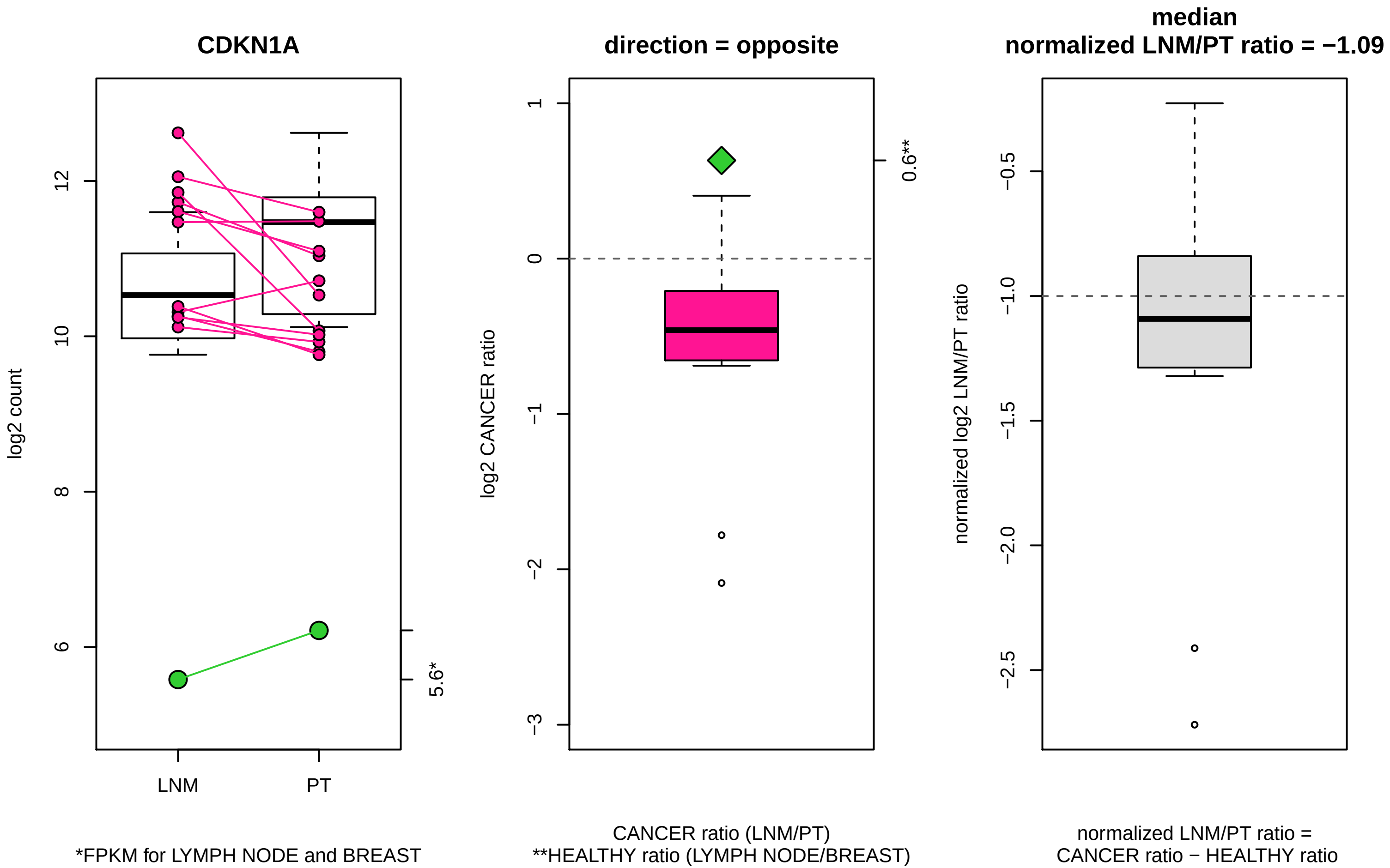

CDKN1A
direction = opposite
median
normalized LNM/PT ratio = −1.09
log2 count
12
10
8
6
5.6*
LNM
PT
*FPKM for LYMPH NODE and BREAST
log2 CANCER ratio
1
0
−1
−2
−3
0.6**
CANCER ratio (LNM/PT)
**HEALTHY ratio (LYMPH NODE/BREAST)
normalized log2 LNM/PT ratio
−0.5
−1.0
−1.5
−2.0
−2.5
normalized LNM/PT ratio =
CANCER ratio − HEALTHY ratio

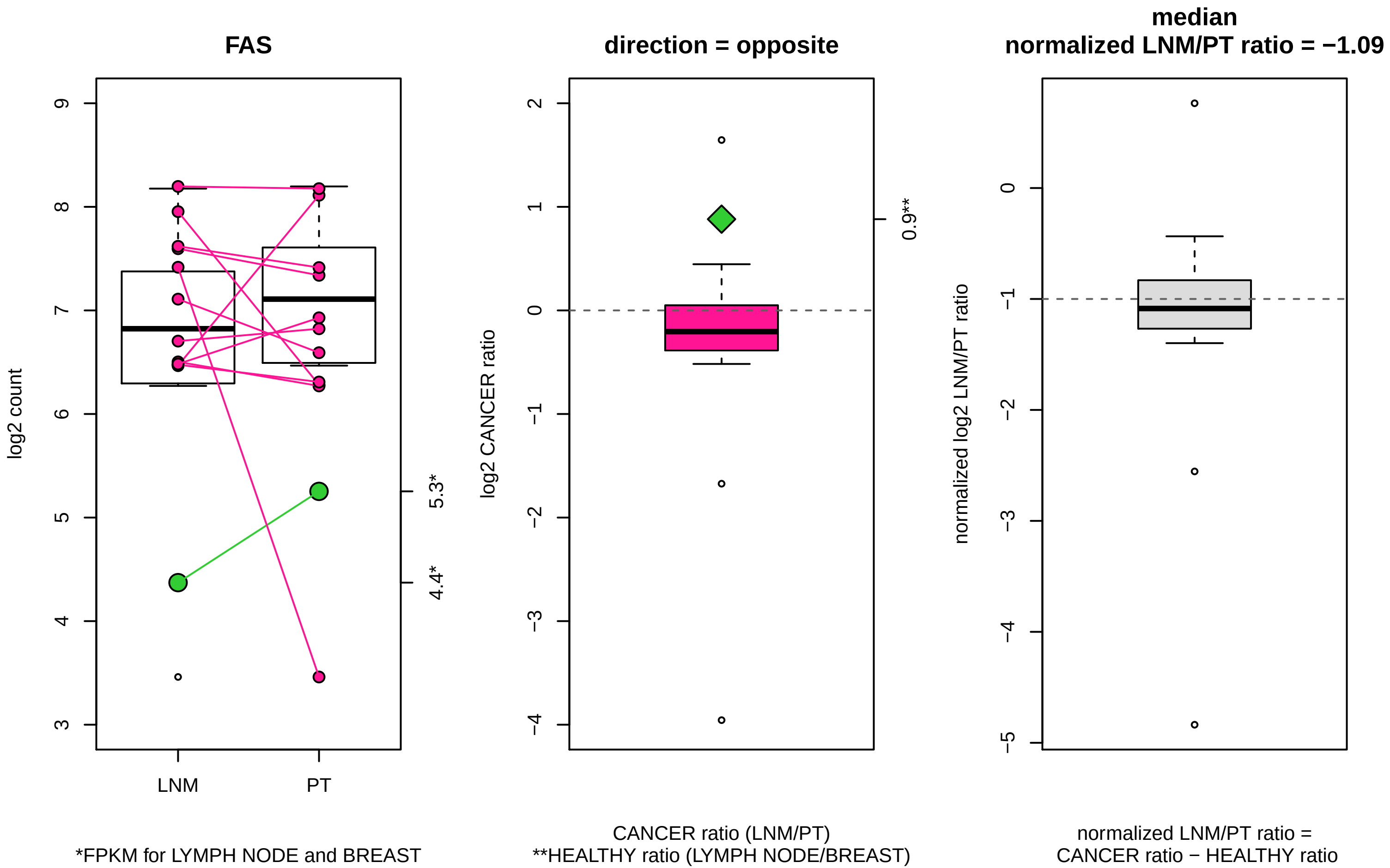
FAS
log2 count
9
8
7
6
5
4
3
LNM
PT
5.3*
4.4*
*FPKM for LYMPH NODE and BREAST
direction = opposite
log2 CANCER ratio
2
1
0
−1
−2
−3
−4
0.9**
CANCER ratio (LNM/PT)
**HEALTHY ratio (LYMPH NODE/BREAST)
median
normalized LNM/PT ratio = −1.09
normalized log2 LNM/PT ratio
0
−1
−2
−3
−4
−5
normalized LNM/PT ratio =
CANCER ratio − HEALTHY ratio
