# Supplementary figures and images for: Reduced expression of innate immunity-related genes in lymph node metastases of luminal breast cancer patients
Source: Sci Rep. 2021 Mar 3;11:5097. doi: 10.1038/s41598-021-84568-0 (PMC7930267; doi:10.1038/s41598-021-84568-0)

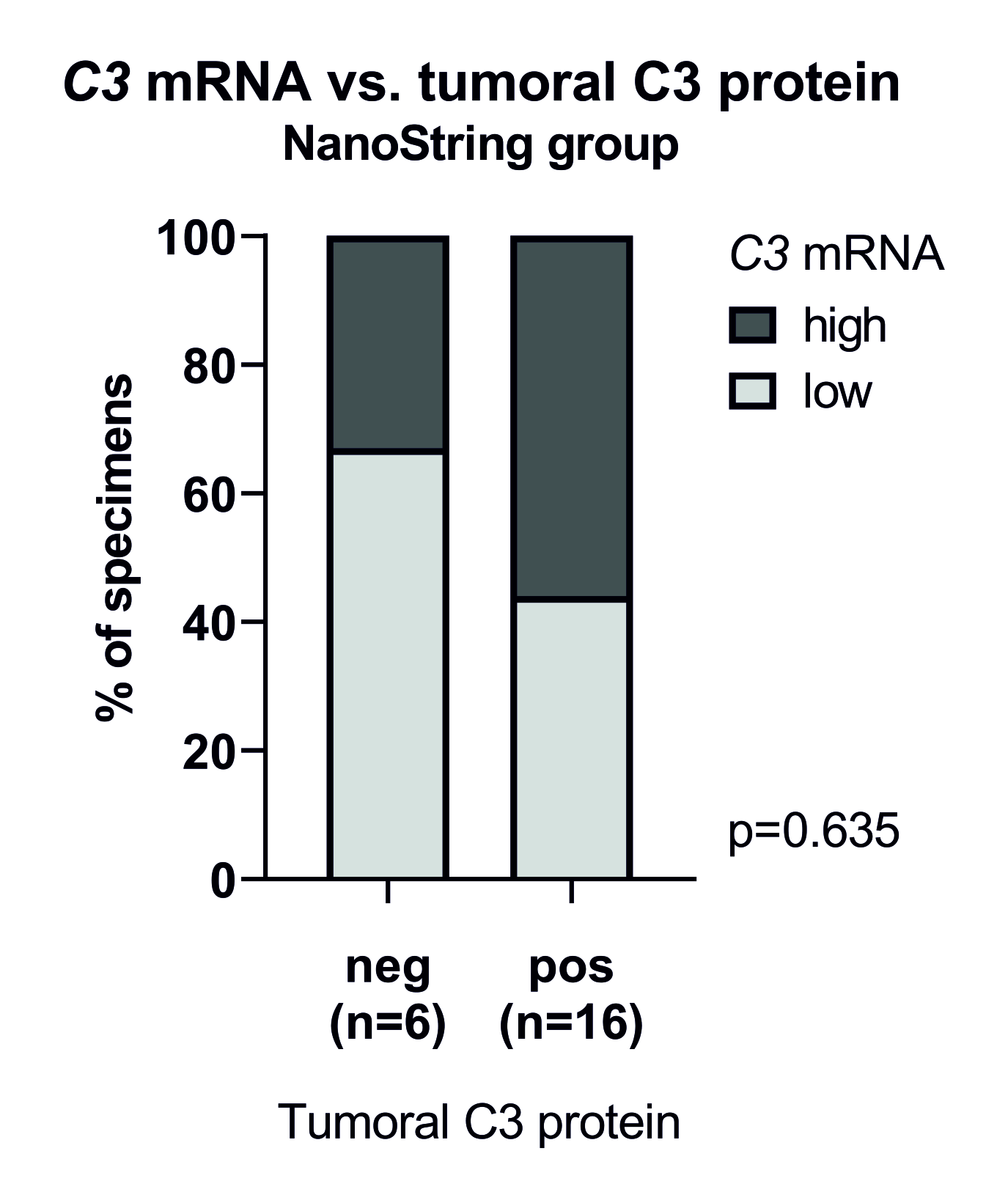

Supplement: Supplementary file 3 — Supplementary Figure S2. [file 41598_2021_84568_MOESM3_ESM.tif]

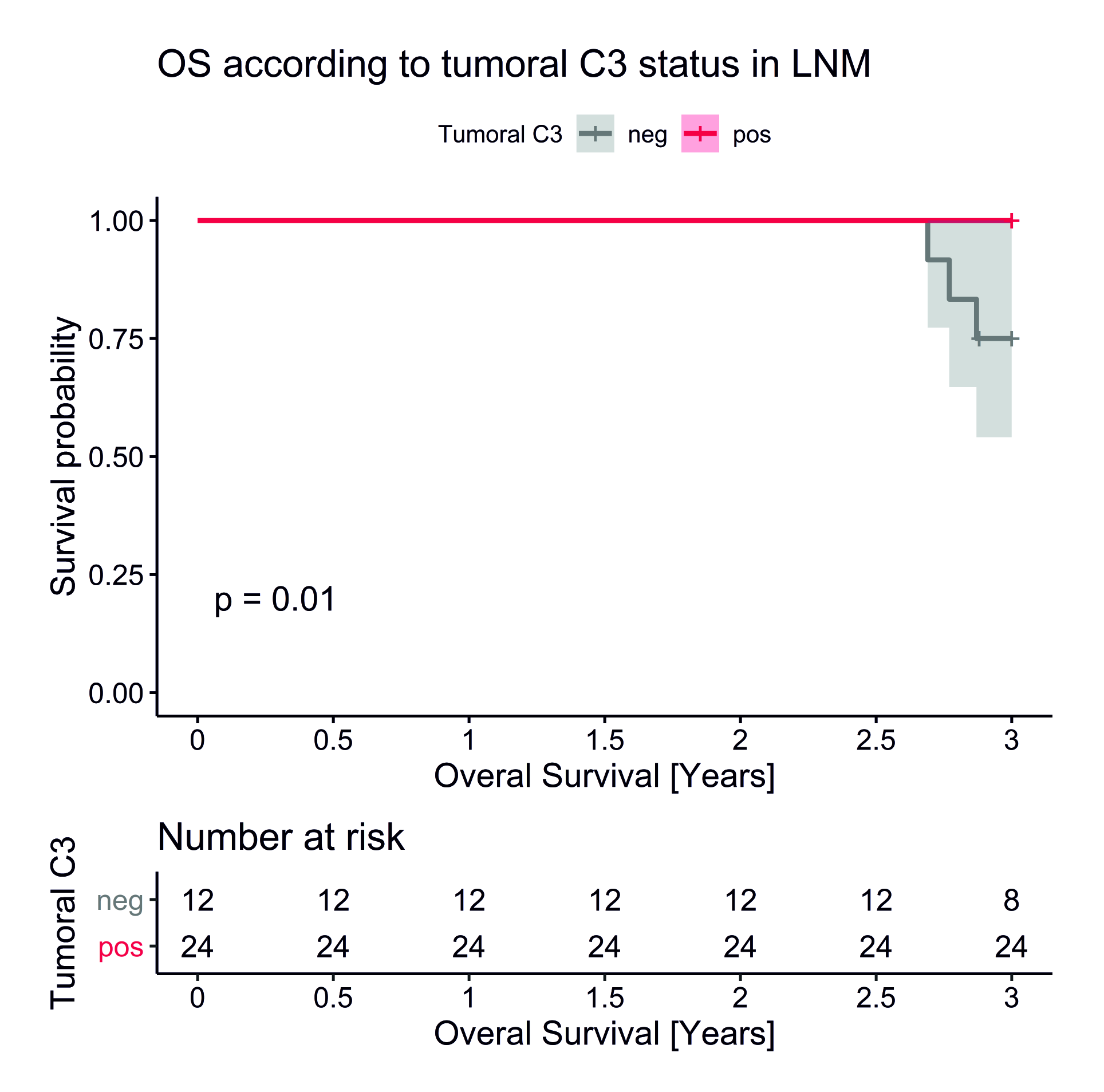

Supplement: Supplementary file 4 — Supplementary Figure S3. [file 41598_2021_84568_MOESM4_ESM.tif]

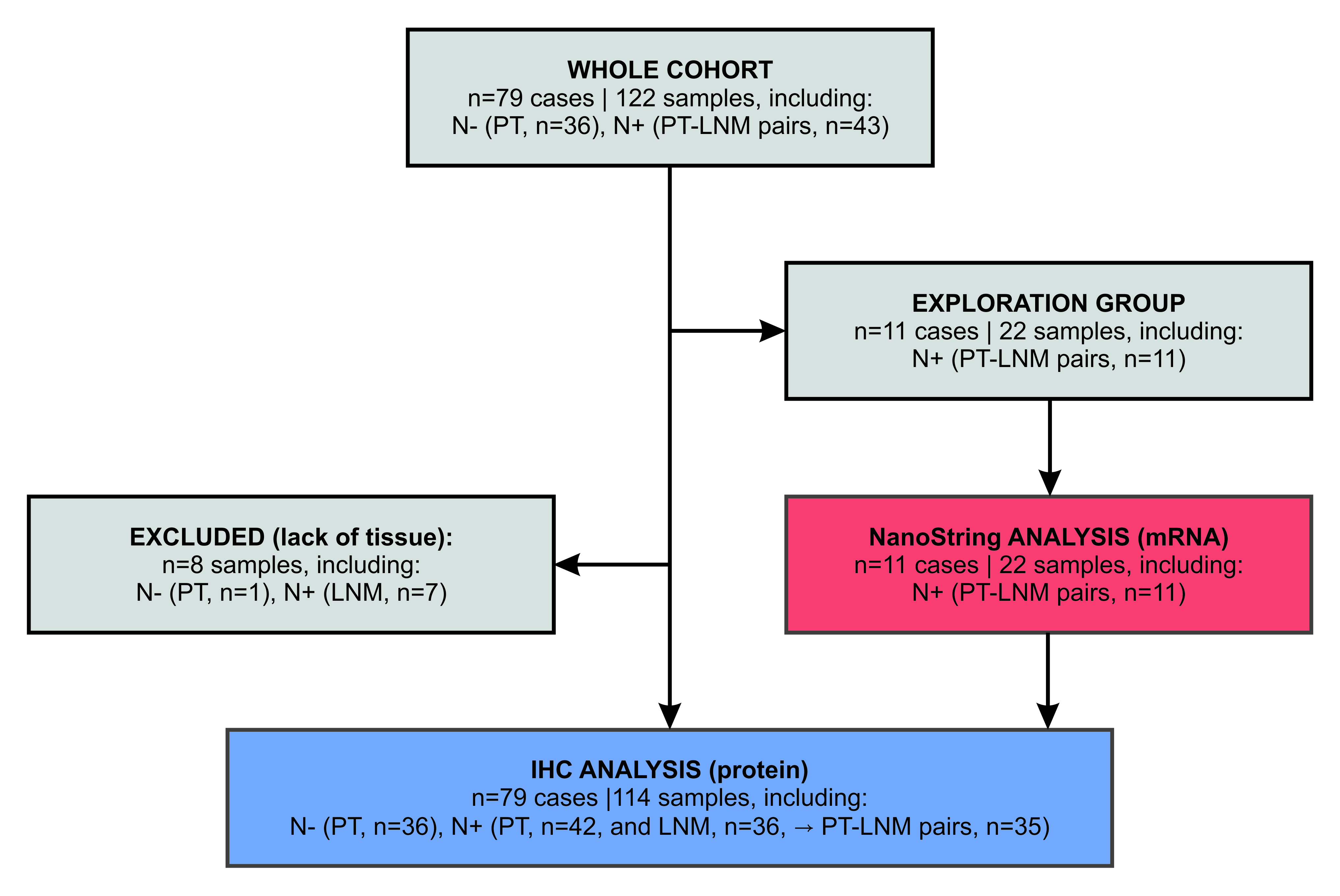

Supplement: Supplementary file 5 — Supplementary Figure S4. [file 41598_2021_84568_MOESM5_ESM.tif]

A

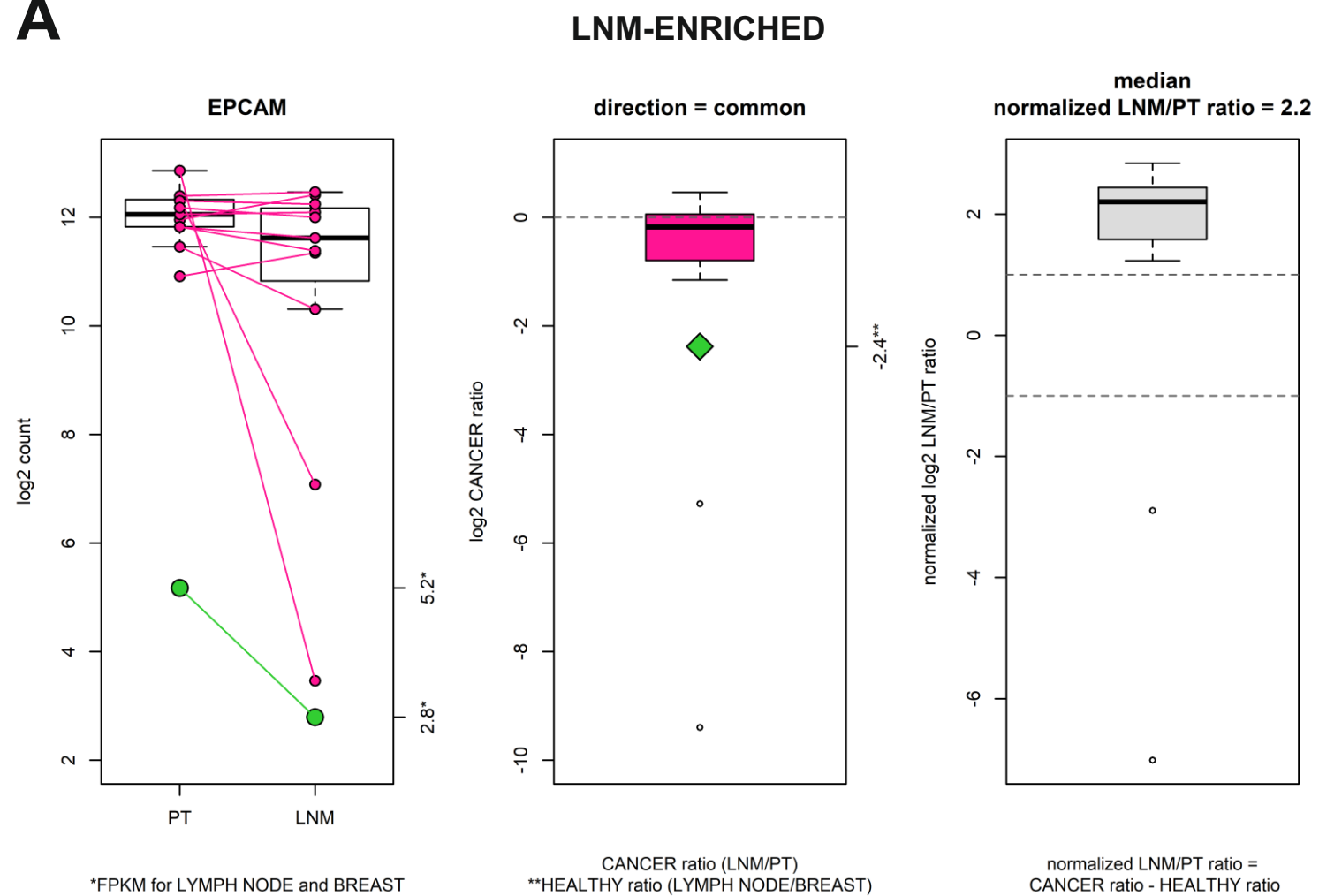

B

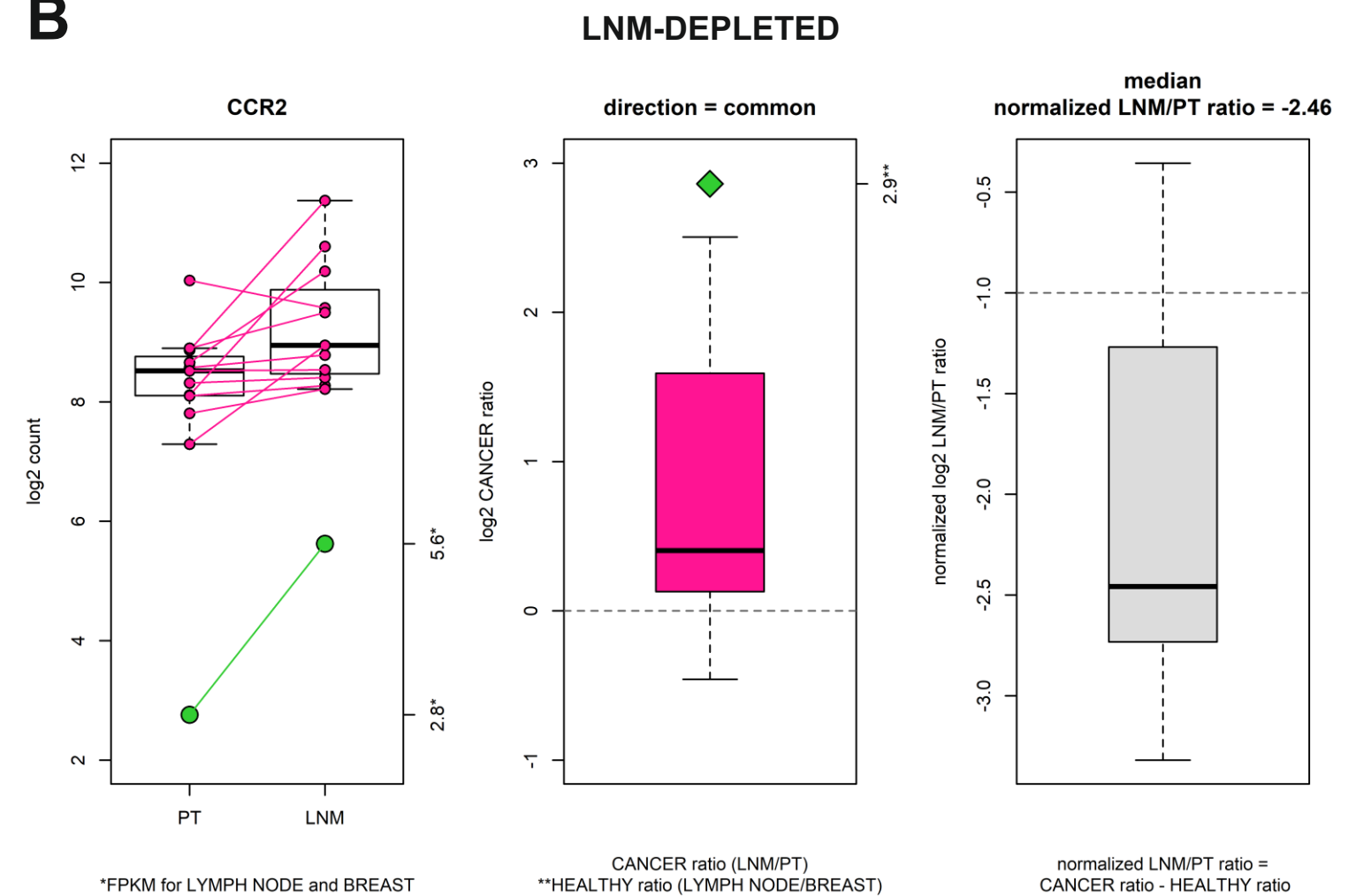

C

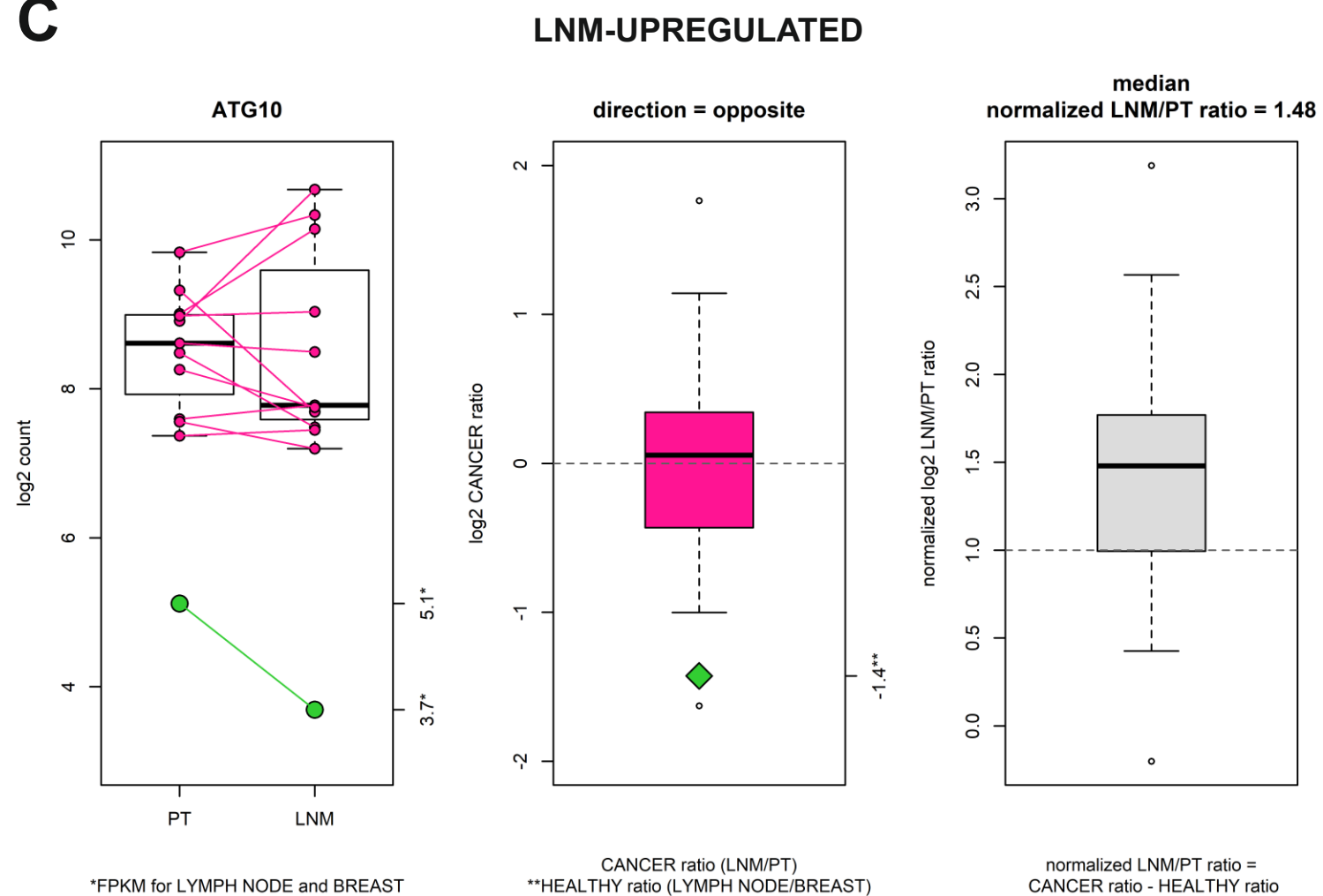

D

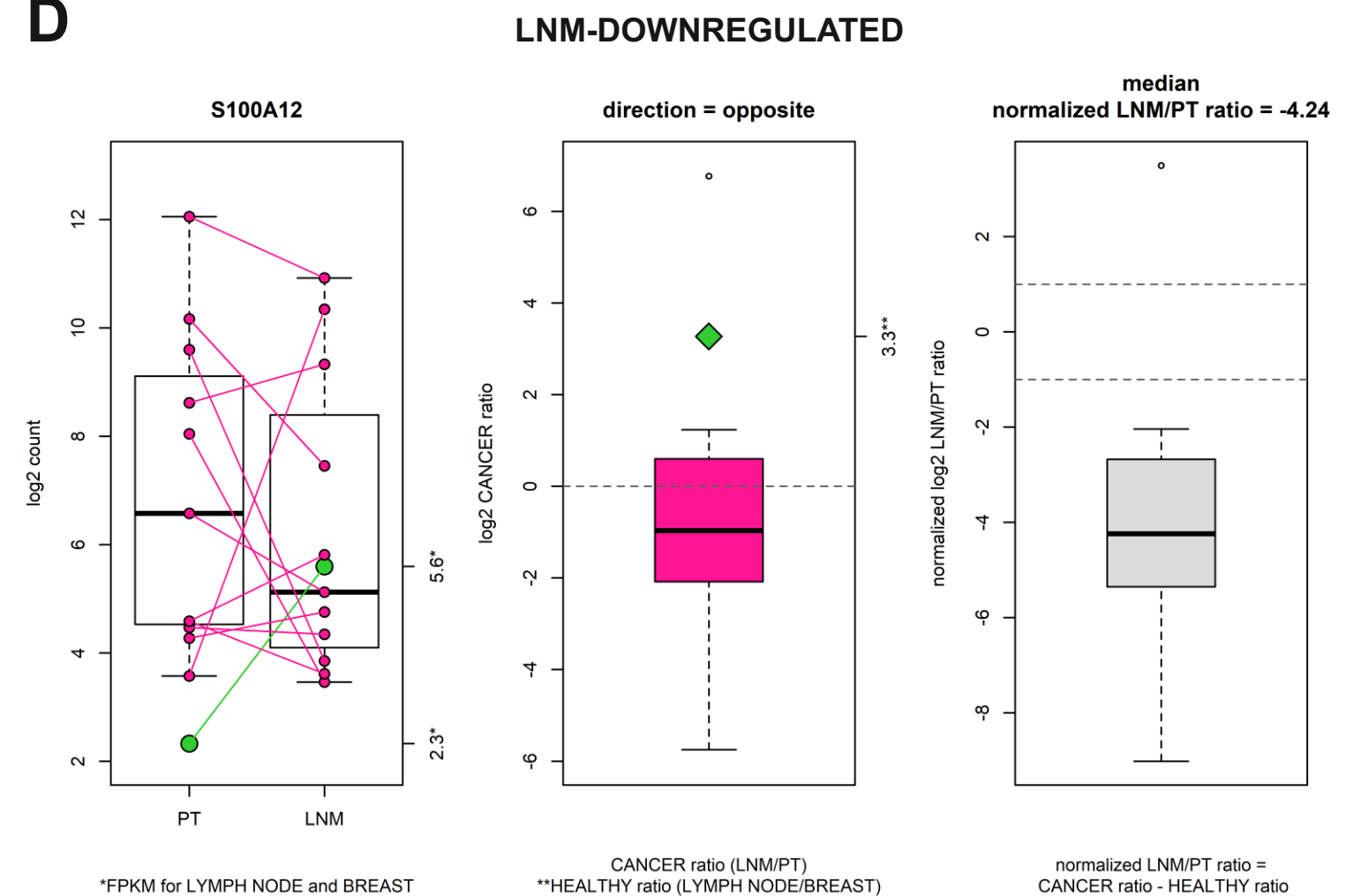

Supplement: Supplementary file 6 — Supplementary Figure S5. [file 41598_2021_84568_MOESM6_ESM.pdf]
